# Transcription factor AP-2α activates RNA polymerase III–directed transcription and tumor cell proliferation by controlling expression of c-MYC and p53

**DOI:** 10.1016/j.jbc.2023.102945

**Published:** 2023-01-25

**Authors:** Juan Wang, Qiyue Chen, Feixia Peng, Shasha Zhao, Cheng Zhang, Xiaoye Song, Deen Yu, Zhongyu Wu, Jiannan Du, Hongwei Ni, Huan Deng, Wensheng Deng

**Affiliations:** 1 School of Life Science and Health, Wuhan University of Science and Technology, Wuhan, China; 2 School of Materials and Metallurgy, Wuhan University of Science and Technology, Wuhan, China

**Keywords:** RNA polymerase III, gene transcription, TFAP2A, cell proliferation, signaling pathways, ChIP, chromatin immunoprecipitation, FLNA, Filamin A, Pol III, RNA polymerase III, RT-qPCR, reverse transcription–quantitative polymerase chain reaction, TFAP2A, transcription factor AP2 alpha

## Abstract

Deregulation of transcription factor AP2 alpha (TFAP2A) and RNA polymerase III (Pol III) products is associated with tumorigenesis. However, the mechanism underlying this event is not fully understood and the connection between TFAP2A and Pol III–directed transcription has not been investigated. Here, we report that TFAP2A functions as a positive factor in the regulation of Pol III–directed transcription and cell proliferation. We found TFAP2A is also required for the activation of Pol III transcription induced by the silencing of filamin A, a well-known cytoskeletal protein and an inhibitor in Pol III–dependent transcription identified previously. Using a chromatin immunoprecipitation technique, we showed TFAP2A positively modulates the assembly of Pol III transcription machinery factors at Pol III–transcribed gene loci. We found TFAP2A can activate the expression of Pol III transcription-related factors, including BRF1, GTF3C2, and c-MYC. Furthermore, we demonstrate TFAP2A enhances expression of MDM2, a negative regulator of tumor suppressor p53, and also inhibits p53 expression. Finally, we found MDM2 overexpression can rescue the inhibition of Pol III–directed transcription and cell proliferation caused by TFAP2A silencing. In summary, we identified that TFAP2A can activate Pol III–directed transcription by controlling multiple pathways, including general transcription factors, c-MYC and MDM2/p53. The findings from this study provide novel insights into the regulatory mechanisms of Pol III–dependent transcription and cancer cell proliferation.

Transcription factor AP2 alpha (TFAP2A) is a member of the activator protein 2 transcription factor (TFAP2) family that plays vital roles in tissue and organ development, apoptosis, gene expression, cell proliferation, and carcinogenesis. TFAP2 factors regulate gene expression by binding to the promoters of its target genes containing AP2 binding sites (GCC(N)3/4GGC) (1, 2, 3, 4). The TFAP2 factors not only modulate the expression of protein-coding genes such as *p21*, *ER-α*, and *HER2* (5, 6, 7) but also control the expression of long noncoding RNA genes, including lncRNA *GAS5* and *SLC2A1-AS1* genes (8, 9). TFAP2A activity can be modulated by posttranslational modification such as phosphorylation and SUMOylation (3, 10, 11). Furthermore, TFAP2A regulates a range of cellular processes by interacting with diverse functional factors, including Sp1, p53, c-MYC, Ying Yang 1 (YY1), retinoblastoma protein, and WW domain–containing oxidoreductase (3, 12, 13, 14, 15, 16, 17). It has been shown that TFAP2A regulates tumor growth, metastasis, and cancer progression by interacting with noncoding lncRNAs (8, 9, 18) and miRNAs (19, 20, 21, 22). Although TFAP2A has been described to be a tumor suppressor in some cancer types (4, 23, 24, 25), it can also act as an oncogenic factor to promote cancer cell proliferation and metastasis (4, 26, 27, 28). For example, the AP-2α/Elk-1 signaling has been reported to control the Sirpα-dependent colorectal cancer cell phagocytosis by regulating tumor-associated macrophages (29). MiR-204-5p and TFAP2A can form a feedback loop that activates cervical cancer proliferation, migration, invasion, and epithelial to mesenchymal transition (30). In lung cancers, TFAP2A potentiates lung adenocarcinoma metastasis by regulating the miR-16 family/TFAP2A/PSG9/TGF-beta signaling pathway (31). The zinc-finger protein 471 was shown to inhibit gastric cancer progression through transcriptionally repressing downstream PLS3 and TFAP2A, suggesting that TFAP2A and PLS3 act as oncogenic factors in gastric cancer cell development (32). Despite enormous advances achieved in cancer development, the mechanism by which TFAP2A promotes cancer development has yet to be elucidated.

RNA polymerase III (Pol III) products are a subset of abundant noncoding RNA molecules, including 5S rRNA, tRNA, U6 snRNA, and 7SL RNA. These RNAs, accounting for about 15% of transcription activity in eukaryotes, play essential roles in several fundamental cellular activities that include ribosomal assembly, translation, protein transportation, RNA processing, and cell growth (33, 34, 35, 36). Pol III–directed transcription initiation can be classified into three types of initiation modes and is tightly controlled by general transcription factors such as TFIIIA, TFIIIB, TFIIIC, and proximal sequence element-binding factor SNAPc (33, 34). Other factors, including MAF1, oncogenic factor c-MYC, tumor suppressors (p53 and retinoblastoma protein), and signaling factors (ERK, JNK, and PTEN), also modulate Pol III–mediated transcription by directly or indirectly interacting with general transcription factors (33, 37, 38, 39, 40, 41). It has been shown that the synthesis of Pol III products can be regulated by chromatin modification (35, 42, 43). For example, *nc886* is a typical gene transcribed by Pol III and its expression is inhibited by CpG methylation (44). Deregulation of Pol III transcription-related factors or Pol III products is associated with a range of diseases such as neural degeneration diseases and cancers (36, 45, 46, 47). Our previous study revealed that cytoskeletal Filamin A (FLNA) can inhibit Pol III–dependent transcription in transformed cell lines (48). However, the factors that participate in this process remain to be identified. In this study, we showed that TFAP2A not only activates Pol III–directed transcription but also is required for the inhibition of Pol III–dependent transcription mediated by FLNA. We then elucidated the regulatory mechanisms of Pol III transcription mediated by TFAP2A.

## Results

### FLNA inhibits TFAP2A expression

**Figure 1:**
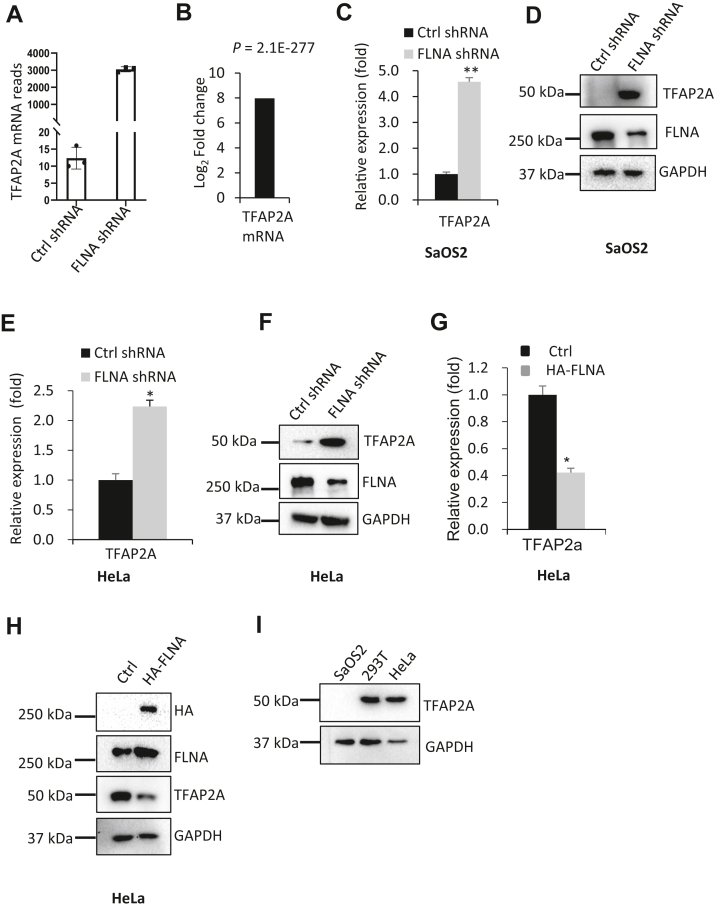
**FLNA inhibits TFAP2A expression in both SaOS2 and HeLa cells.***A* and *B*, effect of FLNA silencing on TFAP2A mRNA expression was analyzed using the mRNA-Seq data obtained from SaOS2 cell lines expressing control shRNA or FLNA shRNA. TFAP2A mRNA reads from three biological replicates and the log_2_ fold change are presented in *A* and *B*, respectively. Log_2_ fold change was obtained by comparing the mean of TFAP2A mRNA reads from FLNA shRNA samples with that from Ctrl shRNA samples. *C* and *D*, effect of FLNA knockdown on TFAP2A expression in SaOS2 cells; RT-qPCR (*C*) and Western blot (*D*) were performed using SaOS2 cells stably expressing control shRNA or FLNA shRNA. *E* and *F*, effect of FLNA knockdown on TFAP2A expression in HeLa cells. RT-qPCR (*E*) and Western blot (*F*) were performed using HeLa cell lines stably expressing control shRNA or FLNA shRNA. *G* and *H*, effect of FLNA overexpression on TFAP2A expression in HeLa cells. A HeLa cell line stably expressing HA-FLNA and its control cell line (empty vector) were used to extract total RNA. TFAP2A mRNA expression was analyzed by RT-qPCR using the cDNA synthesized from the total RNA (*G*). TFAP2A protein expression was detected by Western blot using the total cell lysate of these cell lines and the antibodies against the factors as indicated (*H*). *I*, expression of TFAP2A in SaOS2, 293T, and HeLa cells was analyzed by Western blot. Each column in histograms represents the mean ± SD of three biological replicates. ∗*p* < 0.05; ∗∗*p* < 0.01. *p* values were obtained by Student’s *t* test. RT-qPCR, reverse transcription–quantitative polymerase chain reaction.

In our previous work, we found that cytoskeletal FLNA suppresses Pol III–dependent transcription in human transformed cell lines (48). To understand the regulatory mechanisms underlying this event, we recently performed RNA-Seq analysis using SaOS2 cell lines stably expressing FLNA shRNA or control shRNA (SRA accession number: SRP318361, https://www.ncbi.nlm.nih.gov/Traces/study/?acc=PRJNA726417). Based on the RNA-Seq data, we analyzed differential expression genes between two groups of samples and found that TFAP2A mRNA reads were drastically enhanced by FLNA depletion (Fig. 1, *A* and *B*). In addition, we found that FLNA knockdown in SaOS2 cells severely affected cell shapes of SaOS2 but did not affect the shapes of SaOS2 nuclei and nucleoli (Fig. S1), suggesting that cytoskeletal FLNA plays a key role in the maintenance of cell shape. Next, we verified TFAP2A expression by reverse transcription–quantitative polymerase chain reaction (RT-qPCR) and Western blot using SaOS2 cell lines stably expressing FLNA shRNA and control shRNA. Indeed, TFAP2A expression was enhanced by FLNA depletion in either mRNA or protein levels (Fig. 1, *C* and *D*). To determine whether this result can be reproduced in other cell types, we generated HeLa cell lines stably expressing FLNA shRNA and control shRNA using FLNA-expressing lentiviral particles, and TFAP2A expression was assessed by RT-qPCR and Western blot using the resulting cell lines. As expected, FLNA silencing enhanced TFAP2A expression at both mRNA and protein levels (Fig. 1, *E* and *F*). This result is in agreement with that obtained from SaOS2 cells, suggesting that FLNA can inhibit TFAP2A expression. To confirm the inhibitory role of FLNA in TFAP2A expression, we generated a HeLa cell line with FLNA overexpression and its control cell line using a lentiviral transduction system. TFAP2A expression was analyzed by RT-qPCR and Western blot. We showed that FLNA overexpression reduced the expression of TFAP2A mRNA and protein in HeLa cells (Fig. 1, *G* and *H*). Taken together, these results indicate that FLNA may repress TFAP2A expression in SaOS2 and HeLa cells.

### TFAP2A activates Pol III–directed transcription

**Figure 2:**
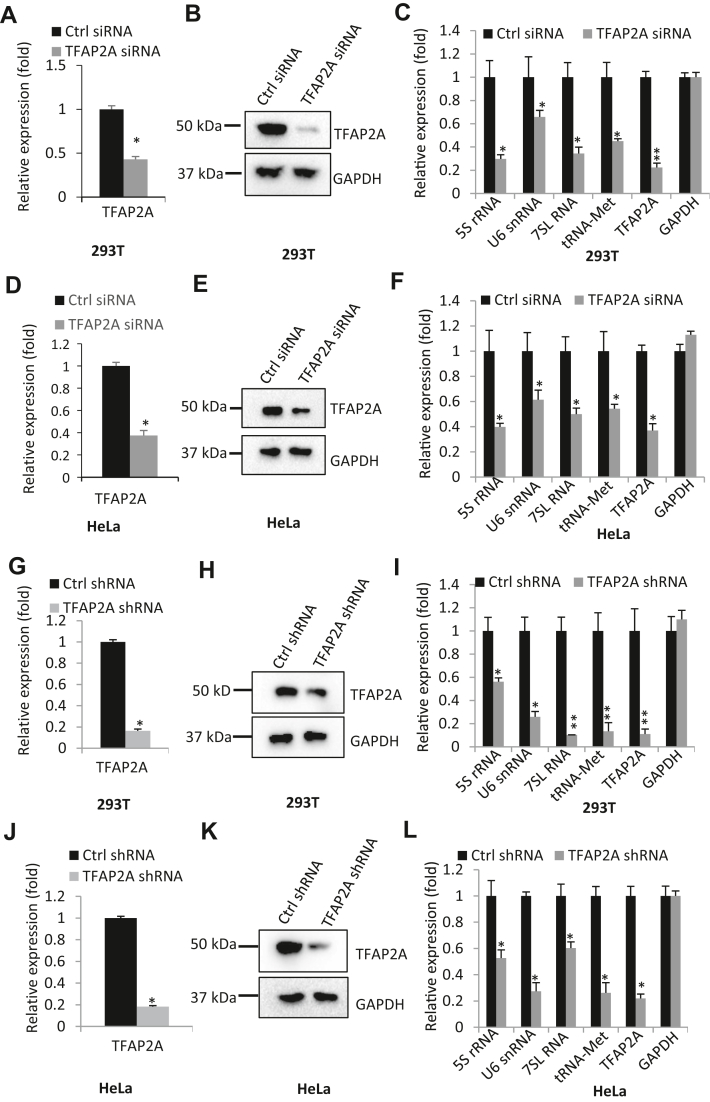
**TFAP2A silencing inhibited the expression of Pol III products.***A*–*C*, transient transfection of TFAP2A siRNA decreased the expression of Pol III products in 293T cells. 293T cells transiently transfected with control siRNA or FLNA siRNA were used to analyze TFAP2A expression by RT-qPCR (*A*) and Western blot (*B*). Pol III products were analyzed by RT-qPCR (*C*). *D*–*F*, transient transfection of TFAP2A siRNA decreased the expression of Pol III products in HeLa cells. HeLa cells transiently transfected with control siRNA or FLNA siRNA were used to analyze TFAP2A expression by RT-qPCR (*D*) and Western blot (*E*). Pol III products were detected by RT-qPCR (*F*). *G*–*I*, TFAP2A shRNA stable expression reduced the expression of Pol III products in 293T cells. 293T cell lines stably expressing control shRNA or FLNA shRNA were used to analyze TFAP2A expression by RT-qPCR (*G*) and Western blot (*H*), where Pol III products were detected by RT-qPCR (*I*). *J*–*L*, TFAP2A shRNA stable expression reduced the expression of Pol III products in HeLa cells. HeLa cell lines stably expressing control shRNA or FLNA shRNA were used to analyze TFAP2A expression by RT-qPCR (*J*) and Western blot (*K*), where Pol III products were detected by RT-qPCR (*L*). Each column in histograms represents the mean ± SD of three biological replicates. ∗*p* < 0.05; ∗∗*p* < 0.01. *p* Values were obtained by Student’s *t* test. RT-qPCR, reverse transcription–quantitative polymerase chain reaction.

Given that FLNA inhibits both Pol III–directed transcription and TFAP2A expression, we asked whether there is a connection between TFAP2A and Pol III–dependent transcription. Before answering this question, we initially investigated TFAP2A expression in several cell types, including SaOS2, 293T, and HeLa cell lines, by immunoblotting. Unexpectedly, TFAP2A expression level in SaOS2 cell line was very low and hard to detect by Western blot, but expression of TFAP2A in 293T and HeLa was quite abundant (Fig. 1, *D* and *I*). Thus, we next focused on the role of TFAP2A in Pol III–directed transcription in HeLa and 293T cell lines. The effect of TFPA2A depletion on the synthesis of Pol III products was first investigated by transfecting TFAP2A siRNA into 293T cells. RT-qPCR and Western blot analysis confirmed TFPA2A downregulation after transient transfection of TFAP2A siRNA (Fig. 2, *A* and *B*). Interestingly, TFAP2A depletion significantly inhibited the synthesis of Pol III products, including 5S rRNA, U6 RNA, 7SL RNA, and tRNA-Met (Fig. 2*C*). Meanwhile, we tested the effect of TFAP2A siRNA transfection on the expression of TFAP2A and Pol III products in HeLa cells; and the results are consistent with those obtained in the assays with 293T cells (Fig. 2, *D*–*F*), indicating that TFAP2A is required for maintaining the normal synthesis of Pol III products. To confirm this result, we generated 293T cell lines that stably express TFAP2A shRNA or control shRNA. As expected, TFAP2A shRNA stable expression dampened TFAP2A expression and the synthesis of Pol III products (Fig. 2, G–I). Consistent results were also achieved when HeLa cells with TFAP2A silencing were analyzed (Fig. 2, *J*–*L*). These results suggest that TFAP2A plays a positive role in Pol III–directed transcription. To validate the positive role of TFAP2A in Pol III–dependent transcription, we assessed the effect of TFAP2A overexpression on Pol III products by performing transient transfection with 239T cells. Notably, TFAP2A overexpression stimulated the expression of Pol III products in 293T cells (Fig. S2, *A* and *B*). Comparable results were obtained when HeLa cells transiently transfected were analyzed (Fig. S2, *C* and *D*). Next, 293T and HeLa cell lines with HA-TFAP2A stable expression were generated and similar experiments were performed. As expected, stable expression of HA-TFAP2A enhanced the expression of Pol III products in both cell types (Fig. S2, *E*–*H*). Altogether, we showed that TFAP2A can positively regulate Pol III–directed transcription.

### Alteration of Pol III products induced by TFAP2A contributes to cell proliferative activity

**Figure 3:**
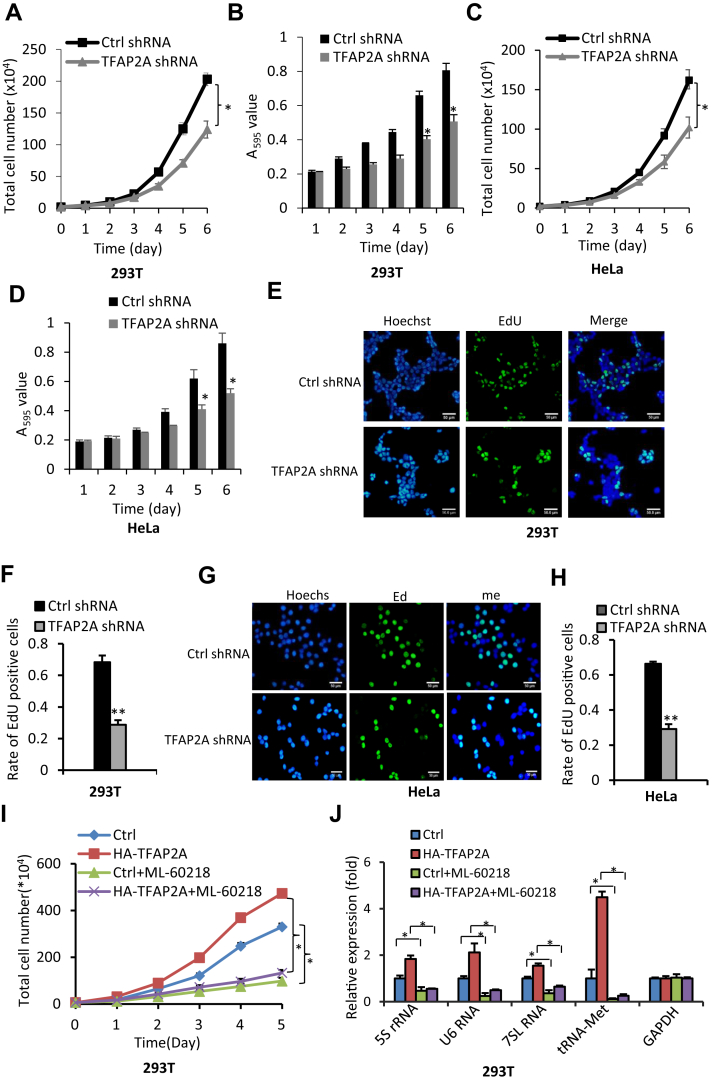
**Effect of TFAP2A expression change on cell proliferation activity.***A* and *B*, TFAP2A knockdown reduced 293T cell proliferation. The proliferative activity of 293T cells stably expressing control shRNA or TFAP2A shRNA was analyzed by cell counting (*A*) and MTT assays (*B*). *C* and *D*, TFAP2A silencing decreased HeLa cell proliferation. The proliferative activity of HeLa cells stably expressing control shRNA or TFAP2A shRNA was analyzed by cell counting (*C*) and MTT assays (*D*). *E* and *F*, EdU assay results showing the effect of TFAP2A downregulation on 293T cell growth. The samples of 293T cells from EdU assays were imaged under a fluorescence microscope, and representative images were presented in *E*. The rate of EdU-positive cells (*F*) was calculated based on the images obtained in multiple samples using ImageJ software. *G* and *H*, EdU assay results for HeLa cells stably expressing control shRNA or TFAP2A shRNA. Images (*G*) for EdU-labeled cells were obtained and presented as for *E*. Scale bars in *E* and *G* represent 50 μm. The rate of EdU-positive cells (*H*) was obtained as described in *F*. *I* and *J*, ML-60218 (a Pol III transcription inhibitor) inhibited the activation of proliferative activity and Pol III–directed transcription induced by TFAP2A overexpression. A 293T cell line expressing HA-TFAP2A and its control cell line were cultured and used for the analysis of proliferative activity (*I*) and Pol III products (*J*) in the presence or absence of 54 μM ML-60218. Each column in histograms represents the mean ± SD of three biological replicates. ∗*p* < 0.05; ∗∗*p* < 0.01. *p* Values in *A*–*D* and *I* were obtained by two-way ANOVA, while other *p* values were obtained by Student’s *t* test.

It is well established that Pol III products are essential to ribosomal assembly, protein synthesis, and cell growth. Thus, we determined whether alteration of Pol III products caused by TFAP2A upregulation or downregulation affects cell growth. We first examined the effect of TFAP2A alteration on the proliferative activity by performing cell counting and MTT assays. Noticeably, TFAP2A knockdown reduced the proliferative activity of 293T and HeLa cells (Fig. 3, *A*–*D*). In contrast, TFAP2A overexpression enhanced them in these cell types (Fig. S3, *A*, *B* and *E*). Since EdU compound (5-ethynyl-2′-deoxyuridine) is widely applied to proliferation assays, we next investigated the effect of TFAP2A alteration on cell growth by performing EdU assays. EdU assays revealed that TFAP2A downregulation reduced the rate of EdU-positive cells (Fig. 3, *E*–*H*), whereas TFAP2A upregulation enhanced the rate of EdU-positive cells (Fig. S3, *C*, *D*, *F* and *G*). These data further confirmed that TFAP2A can promote the proliferative activity of 293T and HeLa cells. To understand whether the enhancement of cell proliferation is caused by the activation of Pol III–dependent transcription, we analyzed proliferative activity and Pol III–dependent transcription in the 293T cell line expressing HA-TFAP2A and its control cell line in the presence or absence of 54 μM ML-60218 (a Pol III transcription inhibitor). As shown in Figure 3, I and *J*, TFAP2A overexpression enhanced 293T cell proliferation and expression of Pol III products, which is consistent with the result as described above (Figs. S2*B* and S3, *A* and *B*). Strikingly, the presence of ML60218 reversed the activation of proliferative activity and Pol III–directed transcription induced by TFAP2A overexpression (Fig. 3, I and *J*). Western blot showed that TFAP2A expression was not significantly affected by the treatment with ML-60218 (Fig. S4). These results suggest that the increase of Pol III products induced by TFAP2A overexpression contributes to the enhancement of proliferation activity, although the contribution of other pathways to cell proliferation cannot be excluded.

### TFAP2A regulates the assembly of the Pol III transcriptional machinery at Pol III target loci and the expression of multiple Pol III transcription-related factors

**Figure 4:**
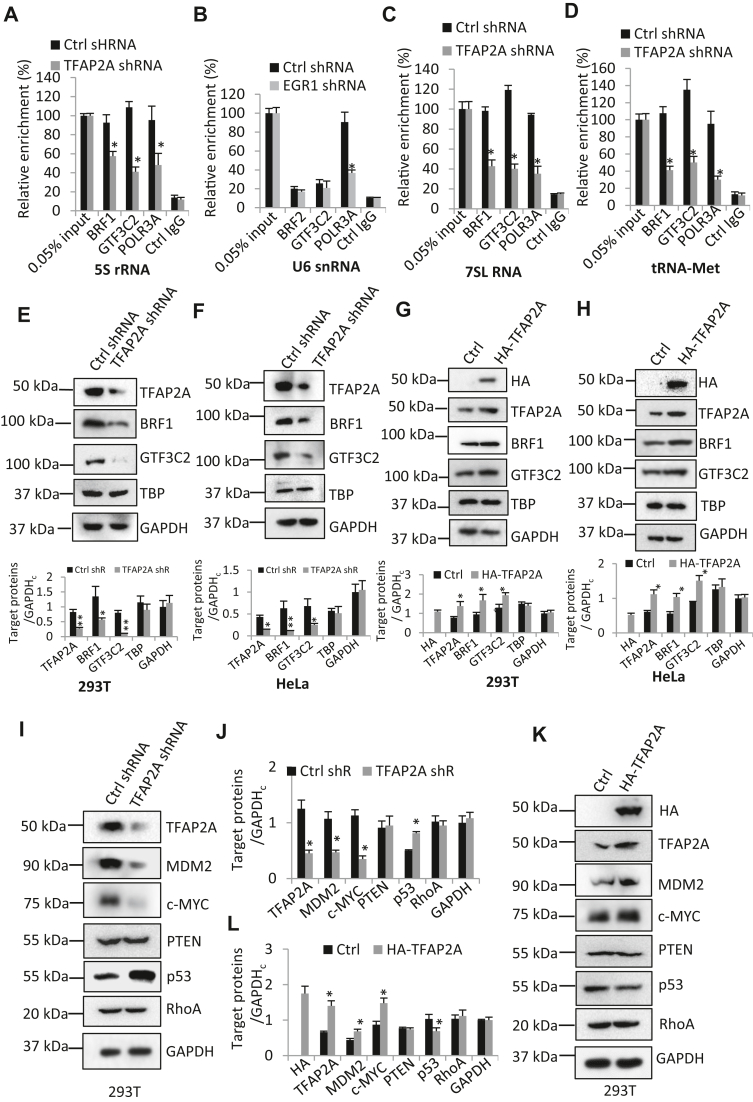
**TFAP2A regulates the recruitment of the Pol III transcription machinery components to Pol III target loci and the expression of Pol III transcription-related proteins.***A*–*D*, TFAP2A silencing inhibited the recruitment of the Pol III transcription machinery components at Pol III target loci. ChIP assays were performed using HeLa cell lines stably expressing control shRNA or TFAP2A shRNA and antibodies against the factors as indicated. Relative enrichment was obtained by comparing the DNA quantity of a gene locus from 1 μl of ChIP DNA samples (1/40 of ChIP DNA) with that from 0.05% input DNA (1 ng genomic DNA). *E* and *F*, TFAP2A downregulation inhibited BRF1 and GTF3C2 expression. Western blot was performed using 293T (*E*) and HeLa (*F*) cell lines stably expressing control shRNA or TFAP2A shRNA and antibodies against the indicated factors. *Bottom panels* in *E* and *F* represent the quantified results of Western blots in the corresponding *upper panels*. GAPDH_c_:GAPDH for control group. *G* and *H*, TFAP2A overexpression enhanced BRF1 and GTF3C2 expression. Western blot was performed using 293T (*G*) and HeLa (*H*) cell lines stably expressing HA-TFAP2A and their control cell lines and antibodies against the indicated factors. *Bottom panels* in *G* and *H* represent the quantified results of Western blots in the corresponding *upper panels*. GAPDH_c_:GAPDH for control group. *I* and *J*, TFAP2A depletion reduced MDM2 and c-MYC expression but increased p53 expression in 293T cells. Western blot was performed as described in *E*. *J*, represents the quantified results of Western blots in I. *K* and *L*, TFAP2A overexpression activated MDM2 and c-MYC proteins but inhibited p53 protein expression in 293T cells. Western blot was performed as described in *G*. *L*, represents the quantified results of Western blots in *K*. Each column in histograms represents the mean ± SD of three biological replicates. ∗*p* < 0.05; ∗∗*p* < 0.01. *p* Values were obtained by Student’s *t* test.

To understand how TFAP2A regulates Pol III–directed transcription, we initially investigated whether TFAP2A binds to the promoters of Pol III target loci by performing chromatin immunoprecipitation (ChIP) assays. The results showed that TFAP2A did not bind to the Pol III target loci except the *5S rRNA* gene locus (Fig. S5*A*). Next, we determined whether TFAP2A silencing affects the assembly of Pol III transcription machinery factors at Pol III target loci by ChIP assays. ChIP analysis showed that TFAP2A silencing reduced the occupancies of components of the Pol III transcription machinery at Pol III target loci, including *5S rRNA*, *7SL RNA*, and *tRNA-Met* loci (Fig. 4, *A*–*D*). BRF1 and GTF3C2 showed very low occupancy on the U6 snRNA gene locus. This result is rational because U6 snRNA transcription does not require the involvement of BRF1 and GTF3C2. However, the occupancy of POLR3A showed significant reduction at this locus. These data suggest that TFAP2A can regulate Pol III–dependent transcription by controlling the recruitment of components of the Pol III transcription machinery to Pol III–transcribed gene loci. To understand how TFAP2A expression alteration affects the assembly of the Pol III transcription machinery at the Pol III–transcribed loci, we analyzed the expression of Pol III transcription-related factors using Western blot and cell lines with TFAP2A deletion or overexpression. It is well established that Pol III general transcription factors play essential roles in Pol III transcription initiation (33). Thus, the expression of Pol III general factor subunits was first analyzed by Western blot using 293T cells with TFAP2A depletion or overexpression. Interestingly, TFAP2A silencing inhibited the expression of BRF1 (one subunit of TFIIIB) and GTF3C2 (one subunit of TFIIIC), but it did not affect TBP expression (Fig. 4*E*). Comparable results were obtained when HeLa cells were analyzed (Fig. 4*F*). In contrast, TFAP2A overexpression activated BRF1 and GTF3C2 expression but did not impact on TBP expression in these cell types (Fig. 4, *G* and *H*). These results suggest that TFAP2A can regulate the assembly of the Pol III transcription machinery at Pol III target loci by affecting BRF1 and GTF3C2 expression.

Pol III–dependent transcription is also controlled by oncogenic factors, tumor suppressors, and signaling transduction factors (33, 34). Next, we investigated whether alteration of TFAP2A expression affects the expression of these factors in 293T and HeLa cells by Western blot. Interestingly, TFAP2A depletion reduced the expression of oncogenic MDM2 and c-MYC; meanwhile, it increased p53 expression in these cell types (Figs. 4, I and *J* and S6, *A* and *B*). Conversely, TFAP2A overexpression enhanced MDM2 and c-MYC expression in which p53 expression was reduced in the same cell types (Figs. 4, *K* and *L* and S6, *C* and *D*). Rho A is an upstream factor of several signal pathways that are required for Pol III–directed transcription (33). In these assays, we found that RhoA expression was not affected by TFAP2A silencing or overexpression (Figs. 4, I and *J* and S6, *A* and *C*). PTEN, a factor in the AKT/PTEN pathway, has been shown to regulate Pol III–directed transcription (41). In these experiments, however, PTEN expression remained unchanged when TFAP2A was upregulated or downregulated in 293T and HeLa cell lines (Figs. 4, I and *J* and S6, *A* and *C*). Taken together, these findings suggest that TFAP2A indirectly regulates Pol III–dependent transcription by affecting the expression of multiple factors, including transcription factor subunits BRF1 and GT3C2, oncogenic c-MYC and MDM2, and tumor suppressor p53.

### TFAP2A regulates Brf1 and Gtf3c2 gene transcription by binding to their promoters

**Figure 5:**
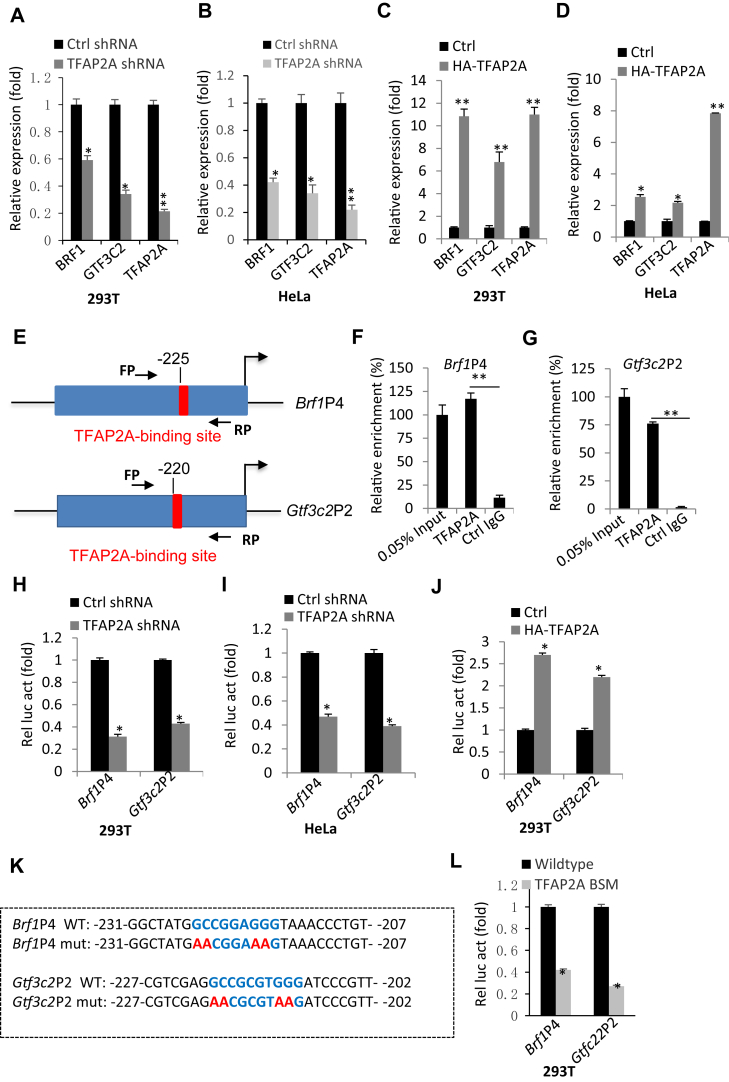
**TFAP2A activates *Brf1* and *Gtf3c2* gene transcription by binding to their promoters.***A* and *B*, TFAP2A knockdown reduced the expression of BRF1 and GTF3C2 mRNA. 293T (*A*) and HeLa (*B*) cell lines stably expressing control shRNA or TFAP2A shRNA were used to analyze the expression of BRF1 and GTF3C2 mRNA by RT-qPCR. *C* and *D*, TFAP2A overexpression enhanced the expression of BRF1 and GTF3C2 mRNA. 293T (*C*) and HeLa (*D*) cell lines stably expressing HA-TFAP2A and the corresponding control cell lines were used to analyze the expression of BRF1 and GTF3C2 mRNA by RT-qPCR. *E*, A diagram showing the location of TFAP2A-binding sites at the *Brf1* promoter 4 (*Brf1*P4) and *Gtf3c2* promoter 2 (*Gtf3c2*P2). The red area within the promoters represents a TFAP2A consensus sequence: GCC(N)3/4GGC/G). FP and RP represent primers used for ChIP-qPCR. FP, forward primer; RP, reverse primer. *F* and *G*, TFAP2A binds to *Brf1*P4 and G*tf3c2*P2. ChIP assays were performed using HeLa cells and the anti-TFAP2A antibody and control IgG. The DNA quantity of *Brf1*P4 (*F*) and G*tf3c2*P2 (*G*) was monitored by qPCR. The relative enrichment was obtained as described in Fig. 4, *A*–*D*. *H* and *I*, TFAP2A depletion by shRNA expression inhibited the activities of *Brf1*P4 and G*tf3c2*P2. Reporter assays were performed by transfecting the *Brf1*P4- or G*tf3c2*P2-driven reporter vectors into 293T (*H*) and HeLa (*I*) cell lines expressing control shRNA or TFAP2A shRNA. Relative luciferase activity (Rel luc act) was obtained by comparing the luciferase activity of treated samples (TFAP2A shRNA) with that of control samples (Ctrl shRNA), where the luciferase activity of the control samples was arbitrarily set as 1. *J*, TFAP2A overexpression activated the activities of *Brf1*P4 and G*tf3c2*P2 in 293T cells. Luciferase assays were performed by transfecting the *Brf1*P4- or G*tf3c2*P2-driven reporter vectors into the 293T cell line expressing HA-TFAP2A and the corresponding control cell line. *K*, a scheme showing the wildtype DNA fragment of *Brf1*P4 and *Gtf3C2*P2 and their mutants containing mutated TFAP2A consensus bases. The *bold blue* bases within the promoter sequences represent wildtype TFAP2A consensus sequence, whereas the bold red bases represent mutated TFAP2A consensus bases (mut). *L*, mutations of TFAP2A consensus sequence severely affected the activities of *Brf1*P4 and G*tf3c2*P2 in 293T cells. Luciferase assays were performed by transfecting 293T cells with the reporter vectors driven by the wildtype promoters (*Brf1*P4 and G*tf3c2*P2) or by their mutants. The relative luciferase activity (Rel luc act) was obtained as described in *H*. BSM, binding site mutations. Each column in histograms represents the mean ± SD of three biological replicates. ∗*p* < 0.05; ∗∗*p* < 0.01. *p* Values were obtained by Student’s *t* test. ChIP, chromatin immunoprecipitation; RT-qPCR, reverse transcription–quantitative polymerase chain reaction.

We showed that TFAP2A expression alteration can affect the expression of BRF1 and GTF3C2 at the protein level. However, how TFAP2A regulates BRF1 and GTF3C2 expression remains to be clarified. To address this question, we first analyzed BRF1 and GTF3C2 mRNA expression in 293T and HeLa cell lines with TFAP2A depletion or overexpression by RT-qPCR. Apparently, TFAP2A knockdown inhibited BRF1 and GTF3C2 mRNA expression, whereas TFAP2A overexpression activated their expression, suggesting that TFAP2A could regulate the expression of *Brf1* and *Gtf3c2* genes at the transcriptional step (Fig. 5, *A*–*D*). To determine whether TFAP2A can modulate the transcription of *Brf1* and *Gtf3c2* genes, we searched TFAP2A-binding sites from the *Brf1 and Gtf3c2 gene* promoters. Surprisingly, the *Brf1* promoter 4 (*Brf1*P4) and the *Gtf3c2* promoter 2 (*Gtf3c2*P2) contain a canonical TFAP2A binding motif (TFAP2A consensus sequence: GCC(N)3/4GGC) (Fig. 5*E*). Next, we examined whether TFAP2A can bind to these two promoters by performing ChIP assays using HeLa cells. ChIP qPCR revealed that TFAP2A displayed specific occupancy at *Brf1*P4 and *Gtf3c2*P2 (Fig. 5, *F* and *G*). Since TFAP2A binds to *Brf1*P4 and *Gtf3c2*P2, we investigated the effect of TFAP2A expression change on the activities of *Brf1*P4 and *Gtf3c2*P2 by performing reporter gene assays. 293T or HeLa cell lines with TFAP2A silencing or overexpression and their corresponding control cell lines were transfected with the reporter gene vectors driven by *Brf1*P4 or *Gtf3c2*P2, respectively. Reporter assays showed that TFAP2A silencing reduced the activities of *Brf1*P4 or *Gtf3c2*P2 (Fig. 5, H and I), whereas TFAP2A overexpression enhanced the activities of these two promoters (Fig. 5*J*). In addition, mutations of TFAP2A-binding sites within these promoters inhibited the activities of *Brf1*P4 or *Gtf3c2*P2 (Fig. 5, *K* and *L*). These results indicate that TFAP2A can directly modulate the transcription of *Brf1* or *Gtf3c2* genes by binding to their promoters. Since the depletion of BRF1 or GTF3C2 has been shown to inhibit Pol III–directed transcription (49), the results obtained from the previous and present experiments suggest that TFAP2A can indirectly activate Pol III–directed transcription by enhancing *Brf1* and *Gtf3C2* gene transcription.

### TFAP2A indirectly regulates Pol III–directed transcription by controlling the activity of the MDM2-p53 pathway

**Figure 6:**
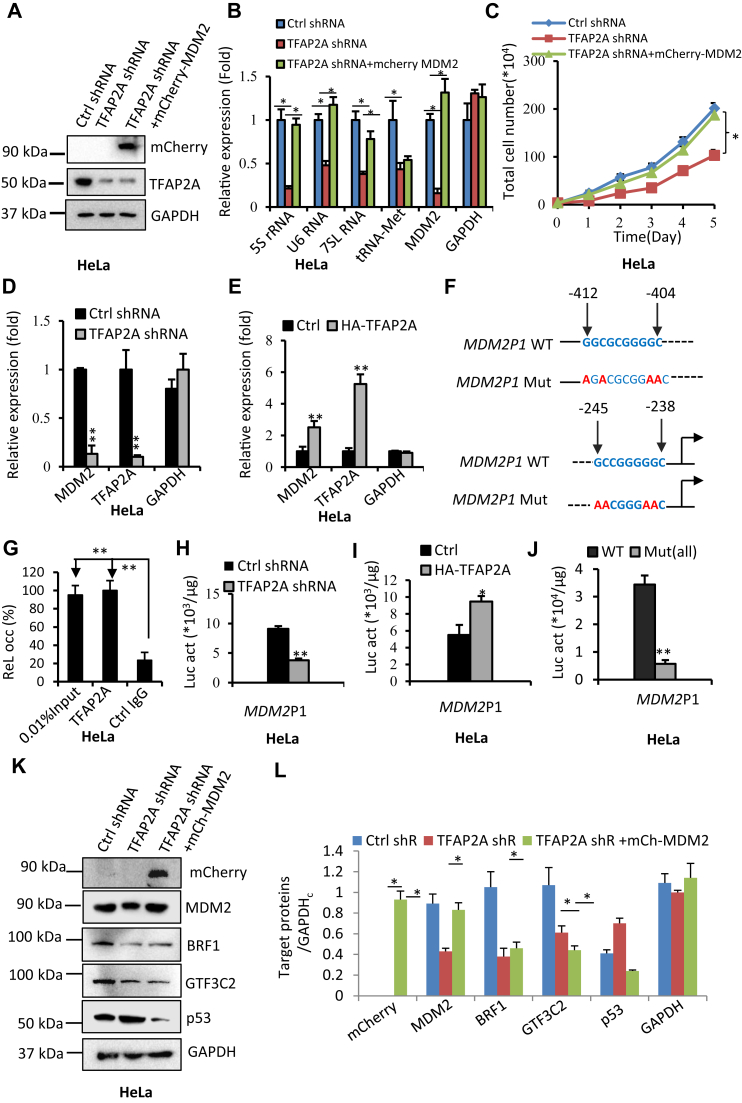
**TFAP2A can modulate Pol III–directed transcription by affecting the activity of the MDM2-p53 pathway.***A*, Western blot results showing the generation of HeLa cell lines expressing both TFAP2A shRNA and mCherry-MDM2. *B*, expression of mCherry-MDM2 reversed the inhibition of Pol III–directed transcription induced by TFAP2A silencing in HeLa cell lines. RT-qPCR was performed using the cell lines established in *A*. *C*, expression of mCherry-MDM2 reversed the inhibition of HeLa cell proliferation caused by TFAP2A silencing. *D* and *E*, RT-qPCR results showing the effect of TFAP2A silencing (*D*) and overexpression (*E*) on MDM2 mRNA expression in HeLa cells. *F*, diagram showing the locations of two TFAP2A consensus sequences (*bold blue letters*) at the *MDM2* promoter 1 and its mutant containing mutated TFAP2A sites (*bold red letters*) in the TFAP2A consensus sequences. *G*, TFAP2A binds to the *MDM2* promoter 1 in HeLa cells. Chromatin immunoprecipitation assays were performed using HeLa cells and an anti-TRFAP2A antibody or control IgG. *H* and *I*, TFAP2A activates the *MDM2*P1 activity in HeLa cells. Luciferase assays were performed by transfecting the *MDM2*P1-driven reporter vectors into HeLa cell lines under TFAP2A silencing (*H*) or overexpression (*I*) and the corresponding cell lines. Luciferase activity for controls or treatments was normalized by the protein quantity used in the assays. *J*, mutations of TFAP2A-binding sites dampened the MDMP1 activity in HeLa cells. Luciferase assays were performed as for *H* and *I*. Mut (all) represents the mutant containing mutations of all TFAP2A-binding sites. *K* and *L*, expression of mCherry-MDM2 inhibited the activation of p53 expression induced by TFAP2A silencing. Western blot was performed using the cell lines generated in *A* and antibodies against the indicated factors. *L*, represents the quantified result of the blots in *K*. GAPDH_c_:GAPDH for control group. Each column in histograms represents the mean ± SD of three biological replicates. ∗*p* < 0.05; ∗∗*p* < 0.01. *p* Values in *C* were obtained by two-way ANOVA, whereas *p* values in other graphs were obtained by Student’s *t* test. RT-qPCR, reverse transcription–quantitative polymerase chain reaction.

We have shown that TFAP2A can regulate the expression of MDM2, p53, and c-MYC in the above experiments, and both c-MYC and p53 have been reported to modulate Pol III–directed transcription (38, 39). To understand how TFAP2A regulates expression of c-MYC and p53, we performed ChIP assays using an anti-TFAP2A antibody and the primers detecting *p53* and *c-MYC* promoters. Unexpectedly, ChIP results showed that TFAP2A did not bind to the promoters of *c-MYC* and *p53* genes (Fig. S5*B*), suggesting that TFAP2A indirectly regulates the expression of c-MYC and p53. We next focused on the role of MDM2 in Pol III–mediated transcription. First, we examined whether MDM2 overexpression can reverse the inhibition of Pol III–directed transcription caused by TFAP2A downregulation. To this end, both 293T and HeLa cell lines stably expressing TFAP2A shRNA were further infected by lentiviral particles expressing mCherry-MDM2, and the generation of cell lines expressing both TFAP2A shRNA and mCherry-MDM2 were verified by Western blot (Figs. 6*A* and S7*A*). We showed that mCherry-TFAP2A expression reversed the inhibition of Pol III–directed transcription caused by TFAP2A silencing in both 293T and HeLa cells (Figs. 6*B* and S7*B*). Interestingly, consistent results were obtained when human cervical epithelial cells expressing TFAP2A shRNA and mCherry-MDM2 were generated and used for similar assays (Fig. S8). Since alteration of Pol III products can affect cell proliferation, we next examined the effect of mCherry-MDM2 expression on the proliferative activity of the cell lines with TFAP2A silencing. Interestingly, the mCherry-MDM2 expression also reversed the inhibition of cell proliferation induced by TFAP2A silencing in 293T and HeLa cell lines (Figs. 6*C* and S7, *C*–*E*). How TFAP2A regulates MDM2 expression remains unclear. To address this question, MDM2 mRNA expression in the cell lines with TFAP2A silencing or overexpression was detected by RT-qPCR. We showed that TFAP2A can activate MDM2 mRNA expression (Figs. 6, *D* and *E* and S7, *F* and *G*), suggesting that TFAP2A regulates MDM2 expression at the transcriptional step. To support this assumption, we searched TFAP2A-binding sites within the *Mdm2* gene promoter. Surprisingly, the *Mdm2* promoter contains two putative TFAP2A-binding sites upstream of transcription starting site (Fig. 6*F*). We next examined whether TFAP2A binds to the *Mdm2* promoter by performing ChIP assays. ChIP-qPCR results showed that TFAP2A exhibited specific occupancy at the *Mdm2* promoter compared with that of control IgG (Fig. 6*G*), indicating that TFAP2A can bind to the *Mdm2* promoter. We next asked whether TFAP2A expression change affects the *Mdm2* promoter activity. To answer this question, we prepared the DNA fragments of the wildtype MDM2 promoter and its mutant containing TFAP2A-binding site mutations by PCR and inserted them into the pGL3-basic reporter vector (Fig. 6*F*). Luciferase assays were performed using the cell lines transiently transfected with the promoter-driven reporter vectors. The data from luciferase activity showed that TFAP2A downregulation inhibited the activity of the *Mdm2* promoter activity, whereas TFAP2A overexpression activated its activity (Figs. 6, H and I and S7, *H* and *I*). Intriguingly, mutations of TFAP2A-binding sites reduced the *Mdm2*P1 activity (Figs. 6*J* and 4J). These data indicate that TFAP2A can regulate *Mdm2* gene transcription by binding to the *Mdm2* promoter. MDM2 was identified to be an inhibitor of p53 (50); moreover, MDM2 expression was opposite to p53 (Fig. 4, I and *J*). Thus, we determined the effect of mCherry-MDM2 expression on p53 expression in 293T and HeLa cell lines with TFAP2A silencing. As expected, mCherry-MDM2 expression inhibited the activation of p53 expression induced by TFAP2A silencing (Figs. 6*K* and S7*K*). However, mCherry-MDM2 expression in the TFAP2A shRNA-expressing cells did not rescue the expression of BRF1 and GTF3C2. Similar results were observed when mCherry-MDM2 was expressed in human cervical epithelial cells with TFAP2A silencing (Fig. S8, *B* and *C*). Collectively, these results indicate that TFAP2A can indirectly regulate Pol III–directed transcription by controlling the MDM2-p53 axis.

### TFAP2A is required for the inhibition of Pol III transcription mediated by FLNA

**Figure 7:**
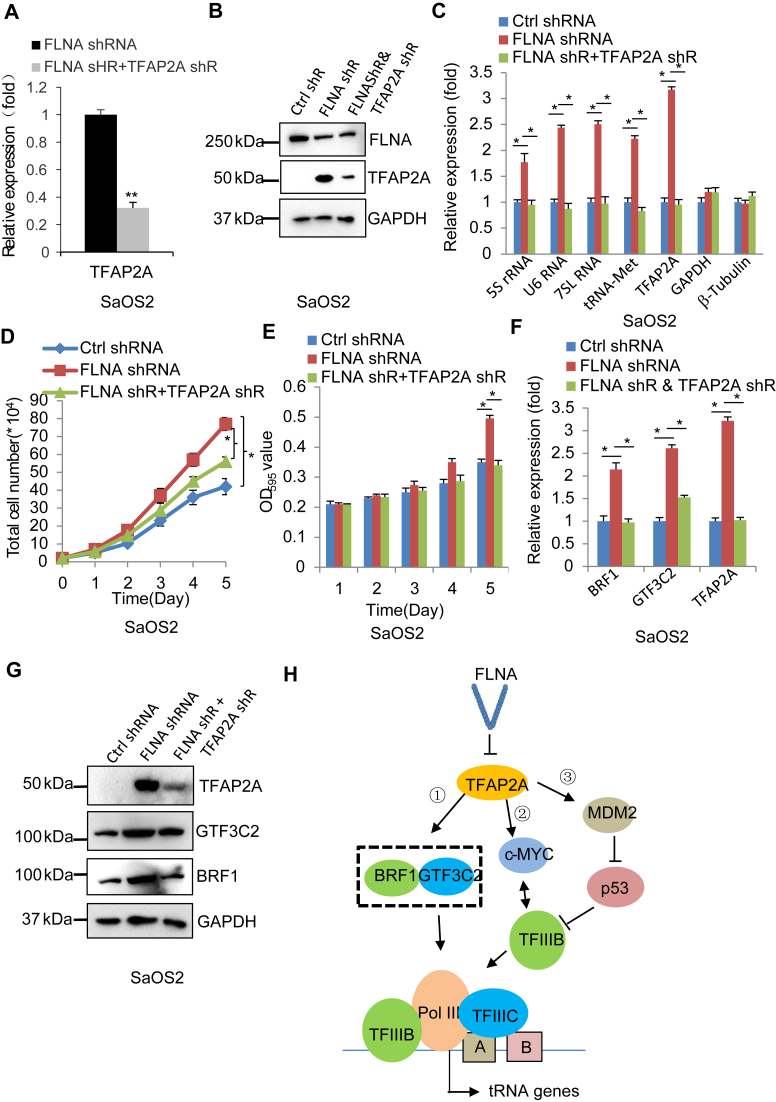
**TFAP2A is required for the activation of Pol III–directed transcription induced by FLNA silencing.***A* and *B*, generation of SaOS2 cell lines expressing both FLNA shRNA and TFAP2A shRNA. TFAP2A expression in the cell lines as indicated was analyzed by RT-qPCR (*A*) and Western blot (*B*). *C*, TFAP2A depletion inhibited the activation of Pol III–directed transcription induced by FLNA silencing in SaOS2 cells. Pol III products were analyzed by RT-qPCR. *D* and *E*, TFAP2A depletion inhibited the enhancement of SaOS2 cell proliferation induced by FLNA silencing in SaOS2 cells. Cell counting (*D*) and MTT (*E*) assays were performed using cell lines established in *A* and *B* and the corresponding control cell line. *F*, BRF1 and GTF3C2 expression in SaOS2 cell lines was detected by RT-qPCR. *G*, BRF1 and GTF3C2 expression in SaOS2 cell lines was detected by Western blot. *H*, a proposed model showing the regulatory mechanisms by which TFAP2A modulates Pol III–directed transcription. Each column in histograms represents the mean ± SD of three biological replicates. ∗*p* < 0.05; ∗∗*p* < 0.01. *p* Values were obtained by Student’s *t* test. RT-qPCR, reverse transcription–quantitative polymerase chain reaction.

Previous work showed that FLNA downregulation enhances Pol III–directed transcription (48); furthermore, FLNA silencing also activated TFAP2A expression in SaOS2 and HeLa cells (Fig. 1). Whether TFAP2A is required for the inhibition of Pol III transcription induced by FLNA remains unclear. To address this question, we generated SaOS2 and HeLa cell lines that stably express both FLNA shRNA and TFAP2A shRNA using cell lines with FLNA depletion. RT-qPCR and Western blot results showed the achievement of these cell lines (Figs. 7, *A* and *B* and S9, *A* and *B*). Analysis of Pol III products by RT-qPCR revealed that TFAP2A silencing inhibited the activation of Pol III–directed transcription induced by FLNA depletion (Figs. 7*C* and S9*C*), indicating that TFAP2A is required for the activation of Pol III transcription induced by FLNA silencing. These data suggest that FLNA and TFAP2A can play opposite roles in Pol III–dependent transcription. FLNA has been shown to suppress tumor cell proliferation (48). Thus, cell proliferation assays were performed using cell lines established above. The results from cell counting and MTT assays revealed that TFAP2A depletion inhibited the enhancement of cell proliferation induced by FLNA silencing (Figs. 7, *D* and *E* and S9, *D* and *E*), suggesting that TFAP2A is required for the inhibition of cell proliferation mediated by FLNA. This result is in agreement with that obtained in the analysis of Pol III products (Figs. 7, *C* and *D* and S9, *C* and *D*). TFAP2A has been shown to regulate Pol III–dependent transcription by controlling BRF1 and GTF3C2 expression (Fig. 5). Thus, we analyzed BRF1 and GTF3C2 expression by RT-qPCR and Western blot using cell lines obtained above (Fig. 7, *A* and *B*). Interestingly, TFAP2A depletion inhibited the activation of BRF1 and GTF3C2 induced by FLNA silencing in SaOS2 and HeLa cells (Figs. 7, *E* and *F* and S9, *E* and *F*). This result is consistent with that obtained using cell lines expressing TFAP2A shRNA only (Fig. 4, *E* and *F*), indicating that TFAP2A modulates FLNA-mediated Pol III transcription by controlling BRF1 and GTF3C2 expression. Taken together, TFAP2A is required for the regulation of Pol III–directed transcription mediated by FLNA.

Based on the data obtained in this study, we proposed a model by which TFAP2A regulates Pol III–dependent transcription. Specifically, TFAP2A expression is inhibited by FLNA; however, TFAP2A upregulation activates the expression of BRF1, GTF3C2, c-MYC, and MDM2. On the one hand, the increase of BRF1 and GTF3C2 expression directly enhances Pol III transcription machinery assembly at target loci to activate the synthesis of Pol III products (Fig. 7*H*-①). On the other hand, the activation of oncogenic c-MYC (Fig. 7*H*-②) and MDM2 (Fig. 7*H*-③) increases the interaction between c-MYC and TFIIIB and decreases the interaction between p53 and TFIIIB, which subsequently activates Pol III–dependent transcription. These regulatory pathways revealed by this study extend the understanding of the mechanism of Pol III–directed transcription.

## Discussion

TFAP2A has been identified for decades as a transcription factor that regulates RNA polymerase II–directed gene transcription (1, 2, 5, 6, 7). In this study, we found a novel role of TFAP2A in Pol III–directed transcription, and TFAP2A positively regulates the synthesis of Pol III products (Figs. 2 and S1). We showed that TFAP2A is also required for the activation of Pol III transcription induced by FLNA silencing (Figs. 7 and S9). Thus, the findings of this study extend the functions of TFAP2A as a transcription factor in human cells. It has been reported that TFAP2A can play negative or positive roles in cancer cell proliferation (4). Especially, the positive role of TFAP2A in cancer cell proliferation was frequently revealed in recent years (4). However, the mechanism underlying the event remains elusive. It is well established that Pol III product levels correlates closely with the rate of cell growth (33, 37), and abnormal high expression of Pol III products was observed in several cancer types (51). We showed that the presence of ML-60218 can reverse the activation of both cell proliferation and Pol III–directed transcription induced by TFAP2A overexpression in 293T cells (Fig. 3, I and *J*), indicating that Pol III–mediated transcription activation contributed to the promotion of cell proliferation by TFPA2A overexpression. These findings provide a novel explanation from about how TFAP2A promotes cancer cell proliferation.

General transcription factors TFIIIB and TFIIIC tightly control transcription initiation directed by Pol III (33, 34). We previously showed that BRF1 and GTF3C2 silencing inhibited Pol III–dependent transcription (49). In this study, we found that TFAP2A can positively regulate the synthesis of Pol III products and the expression of BRF1 and GTF3C2. Furthermore, TFAP2A modulates *Brf1* and *Gtf3c2* gene transcription by binding to the promoters of *Brf1* and *Gtf3c2* genes (Figs. 4 and 5). These results suggest that TFAP2A regulates the expression of Pol III products to control BRF1 and GTF3C2 expression (Fig. 7*H*-①). It has been shown that Specificity protein 1 (Sp1) regulates Pol III–directed transcription by controlling transcription of *Brf1* and *Gtf3C2* genes (49). Recently, we found that GATA4 is required for the regulation of Pol III–directed transcription mediated by Sp1 and can directly control Sp1 expression; therefore, FLNA suppresses Pol III–dependent transcription by the FLNA-GATA4-Sp1 pathway (52). Furthermore, both TFAP2A and Sp1 expression can be negatively regulated by FLNA (Fig. 1). Since the promoters of *Brf1* and *Gtf3C2* genes contain Sp1- and TFAP2A-binding sites (Fig. 5), it is possible that TFAP2A and Sp1 can independently regulate Pol III–directed transcription. Thus, FLNA may suppress Pol III–mediated transcription using several parallel pathways. Oncogenic factor c-MYC and tumor suppressor p53 have been reported to modulate Pol III–directed transcription by interacting with TFIIIB (39, 40). In this study, we found that TFAP2A can positively regulate c-MYC and MDM2 expression but negatively affect p53 expression (Figs. 4 and 6). MDM2 has been identified to be an upstream effector of p53 (50), suggesting that TFAP2A can also indirectly modulate Pol III–mediated transcription by controlling the MDM2/p53 axis. Based on the previous and current findings, we conclude that TFAP2A can indirectly regulate Pol III–directed transcription by controlling multiple pathways (Fig. 7*H*). Moreover, TFAP2A participates in the inhibition of Pol III–directed transcription mediated by FLNA, perhaps, using similar mechanisms. These findings put extra weight on the understanding of the regulatory mechanism of Pol III–dependent transcription mediated by FLNA, where FLNA may modulate Pol III–directed transcription by a complicated regulatory network.

In summary, we found that TFAP2A functions as a positive regulator in Pol III–directed transcription and promotes the proliferative activity of 293T and HeLa cells. TFAP2A is also required for the regulation of Pol III transcription mediated by FLNA. Mechanistically, TFAP2A modulates Pol III–directed transcription by controlling the activities of multiple pathways, including the expression of general transcription factor subunits BRF1 and GTF3C2, c-MYC, and MDM2/p53. Our findings provide novel insights into the regulatory mechanisms of Pol III–directed transcription and cell proliferation.

## Experimental procedures

### Plasmids, cells, and reagents

The lentiviral expression vectors such as pLV-U6-EGFP-Puro, pLV-U6-mCherry-Puro, and pLV-EF1α-EGFP-Puro were purchased from Inovogen Tech Co. FLNA shRNA lentiviral particles (SC-35374-V) and control shRNA lentiviral particles (SC-108080) were obtained from Santa Cruz Biotech. Human cell lines, including SaOS2, HeLa, and 293T, were derived from American Type Culture Collection (ATCC). SaOS2 cells were cultured in McCoy’s 5A complete medium (HyClone), while HeLa and 293T cells were cultured in Dulbecco’s modified Eagle’s medium complete medium (HyClone). DNA and RNA oligonucleotides were purchased from Sangon Biotech. PCR DNA purification and gel extraction kits were obtained from Axygen Co. Biological reagents such as restriction enzymes and transfection reagents were obtained from Thermo Scientific. All chemicals were obtained from Sinopharm Chemical Reagent Co.

### Gene cloning

A *Filamin A* gene was amplified by PCR using the cDNA synthesized with 1 μg of the total RNA extracted from 293T cells. The *HA-Filamin A* fusion gene was generated by PCR using the resulting *Filamin A* gene and inserted into the pcDNA 3.0(+). TFAP2A shRNA-coding DNA fragments were synthesized by Sangon Biotech. After annealing and phosphorylation, DNA fragments were loaded downstream of the U6 promoter at the pLV-U6-mCherry-Puro plasmid. MDM2 cDNA fragments were amplified by PCR and loaded downstream of the *mCherry* gene at the pLV-EF1α-mCherry-Puro plasmid to form a *mCherry-MDM2* fusion gene. A *TFAP2A* gene was amplified by PCR using the cDNA synthesized as described above; thereafter, the *HA-TFAP2A* fusion gene was generated by PCR using the resulting *TFAP2A* gene and loaded downstream of the EF1α promoter at the pLV- EF1α-EGFP-Puro plasmid.

### Transfection and generation of stable cell lines

For TFAP2A siRNA transient transfection, 293T and HeLa cells were seeded in 12-well plates. After 24 h, cells in each well were transfected with 6 pmol/L siRNA fragments and 2 μl of transfection reagent. Forty-eight hours post transfection, cell samples were harvested and used to analyze TFAP2A expression with RT-qPCR and Western blot techniques. For the generation of cell lines stably expressing TFAP2A shRNA or HA-TFAP2A protein, 293T cells cultured in six-well plates were transfected with 3 μg of TFAP2A shRNA or HA-TFAP2A-expressing lentiviral vectors and the packaging plasmids pH1 (2 μg)and pH2 (1 μg). After 48 h, the culture medium containing viruses was collected and filtered with a sterile filter (0.45 μm in diameter). Next, 293T or HeLa cells cultured 12-well plates were incubated with the lentivirus-containing medium for 48 h, selected for 72 h at the final concentration of 10 μg/ml puromycin and screened by diluting the drug-resistant cells into 96-well plates. After expansion, individual cell colonies were transferred into 12-well plates and continually cultured in a complete medium containing 10 μg/ml puromycin. Cell lines with stable expression were verified by RT-qPCR and Western blot. SaOS2 cell lines stably expressing FLNA shRNA or control shRNA were achieved by direct transduction with lentiviral particles (Santa Cruz Biotech). SaOS2 and HeLa cell lines stably expressing both FLNA shRNA and TFAP2A shRNA were obtained by incubating the FLNA shRNA-expressing cells with the medium containing TFAP2A shRNA-expressing lentiviral particles. The HeLa cell line expressing both TFAP2A shRNA and mCherry-MDM2 was generated by incubating the TFAP2A-depleted HeLa cell line with the medium containing mCherry-MDM2-expressing lentiviral particles. After selection, cell lines with shRNA or protein stable expression were verified by Western blot using antibodies against FLNA, TFAP2A, HA, and mCherry.

### RT-qPCR and detection of Pol III products

SaOS2, 293T, and HeLa cell lines expressing FLNA shRNA or TFAP2A shRNA were grown in 12-well plates. At 90% confluence, cells were harvested and total RNA was prepared using an RNA extraction kit (Axygen). Reverse transcription was performed at 42 °C in a 20-μL reaction mixture containing 0.5 μg of RNA and 10 U of reverse transcriptase (New England Lab). After incubating for 1 h, the reaction was terminated by heating the sample for 15 min at 85 °C, followed by diluting it with 80 μl of ddH_2_O. Next, 1 μl of cDNA was used for quantitative PCR (qPCR) in which 20 μl of reaction mixture contained 10 μl of 2 × SYBR Green Master Mix (Vazyme Co) and 5 pmoles of the primers detecting Pol III target genes. Quantitative PCR was performed using a Bio-Rad real-time PCR detection system. The resulting data were processed with CFX Manager 3.1 software (Bio-Rad), where an *Actin* or *GADPH* gene was used as a reference gene.

### Western blot analysis

SaOS2, 293T, and HeLa cell lines were cultured in six-well plates. At 90% confluence, cells were harvested and lyzed using 250 μl of 1 × SDS loading buffer. After heating for 10 min at 100 °C, 20 μl cell lysate was loaded into wells within an SDS-PAGE gel for electrophoresis. After that, immunoblotting was performed using antibodies against the factors, including FLNA (Ab76289, Abcam), TFAP2A (Ab108311, Abcam), HA (H9658, Sigma-Aldrich), GAPDH (RAB0101, Frdbrio), BRF1 (SC-81405, Santa Cruz Biotech), GTF3C2 (SC-81406, Santa Cruz Biotech), c-MYC (SC-40, Santa Cruz Biotech), MDM2 (SC-965, Santa Cruz Biotech), p53 (SC-126, Santa Cruz Biotech), RhoA (SC-418, Santa Cruz Biotech), mCherry (TAG0080, Frdbio), TBP (SC-421, Santa Cruz Biotech), and PTEN (SC7974, Santa Cruz Biotech).

### Cell proliferation assays

Cell proliferation assays were performed using different methods, including cell counting, MTT, CCK8, and EdU. For cell counting and EdU assays, 293T and HeLa cell lines expressing TFAP2A shRNA or HA-TFAP2A and their control cell lines were cultured in 12-well plates. Proliferation assays were performed as described (49, 53, 54). For MTT assays, 293T and HeLa cell lines with TFAP2A knockdown or overexpression and their control cell lines were cultured in 96-well plates. MTT assays were performed as described (49). For CCK8 assays, 293T and HeLa cell lines stably expressing both TFAP2A shRNA and mCherry-AP2A and their control cell lines were cultured in 96-well plates. After culturing for 24 h, 10 μl of CCK8 solution (Vazyme) was added to each well and incubated for 4 h at 37 °C. Absorbance values for individual samples at the 450 nm wavelength were measured using the SpectraMax i3 equipment (Molecular Device).

### Chromatin immunoprecipitation assays

HeLa cell lines stably expressing TFAP2A shRNA or control shRNA were cultured in 10-cm dishes. At 90% confluence, cell lines were fixed for 10 min using 10 ml of 1% formaldehyde freshly prepared with 1 × PBS before adding 1 ml of 2.5 M glycine solution into the medium to quench the fixation. After washing twice with 1 × PBS solution, the fixed cells were lysed with 1 ml ChIP lysis buffer (1% SDS, 10 mM EDTA, 50 mM Tris-HCl [pH8.0]), followed by disrupting for 20 min in an ultrasonicator and centrifuging for 10 min at 12,000 rpm. The supernatant was retained and diluted 7 times with RIPA buffer (0.01% SDS, 1.1% TritonX-100, 1.2 mM EDTA, 16.7 mM Tris-HCl pH 8.0, and 167 mM NaCl). The rest of the procedures for ChIP assays were performed as described (49, 54). Chromatin immunoprecipitation was performed using antibodies against BRF1 (SC-81405, Santa Cruz Biotech), BRF2 (Ab17011, Abcam), GTF3C2 (SC-81406, Santa Cruz Biotech), and POLR3A (SC-292119, Santa Cruz Biotech). After de-cross-linking, DNA from ChIP samples or input samples was recovered using a DNA purification kit (Axygen), where 40 μl of ddH_2_O was used to elute the DNA. One microliter of DNA samples (1/40) from a ChIP assay was used for one qPCR reaction; meanwhile, 1 ng of genomic DNA from input (0.05% input) was used as a positive control during qPCR. The relative enrichment of each Pol III gene locus was obtained by comparing the relative DNA quantity from 1 μl of ChIP sample to that from 1 ng input DNA.

### Reporter assays

The DNA fragments for *Brf1* gene promoter 4, *Gtf3C2* gene promoter 2, and *MDM2* gene promoter 1 were amplified from 293T genomic DNA by PCR. The resulting promoter DNA was inserted into pGL3-basic reporter vector (Promega). 293T and HeLa cells were cultured in 12-well plates for 24 h and were subjected to transient transfection with the *Brf1*P4 or *Gtf3c2*P2-driving reporter vectors and the vectors expressing β-galactosidase, where three biological replicates for treatment or control groups were designed. After 48 h, cell samples were harvested and lysed with 100 μl of lysis buffer provided by a dual-light luciferase detection kit (Promega). Five microliters of cell lysate was used to detect the activities of luciferase and β-galactosidase based on the manual provided by the kit. The luciferase activity of each sample was normalized by the activity of β-galactosidase within the same sample. Relative luciferase activity was obtained by comparing the luciferase activity from the treatment samples to that from the control samples, where the activity of the control sample was arbitrarily set as 1. For reporter assays using MDM2P1-driving reporter vectors, luciferase activity for treatment and control was normalized by the protein quantity used in each assay.

### Statistical analysis

The experiments including RT-qPCR, cell proliferation assays, ChIP assays, and reporter assays were performed in three biological replicates. The mean and standard deviation (SD) for the data from gene expression analysis (RT-qPCR), cell proliferation assays, reporter assays, and ChIP assays (qPCR) were calculated using the GraphPad Prism 6.0 software. Each column or point in all digital graphs in this article represents the mean±SD of three biological replicates (n = 3). *p* Values were obtained by a Student’s *t* test or two-way analysis of variance.

## Data availability

All data were included in main figures in the article and figures in the supplementary file.

## Supporting information

This article contains supporting information (a supplementary file).

## Conflict of interest

The authors declare that they have no conflicts of interest with the contents of this article.
