# Supplementary material for: Transcription factor AP-2α activates RNA polymerase III–directed transcription and tumor cell proliferation by controlling expression of c-MYC and p53
Source: J Biol Chem. 2023 Jan 25;299(3):102945. doi: 10.1016/j.jbc.2023.102945 (PMC9999235; doi:10.1016/j.jbc.2023.102945)
Supplement: Supplementary figures S1-S9 [file mmc1.pdf]

Supplemental figures

Fig S1

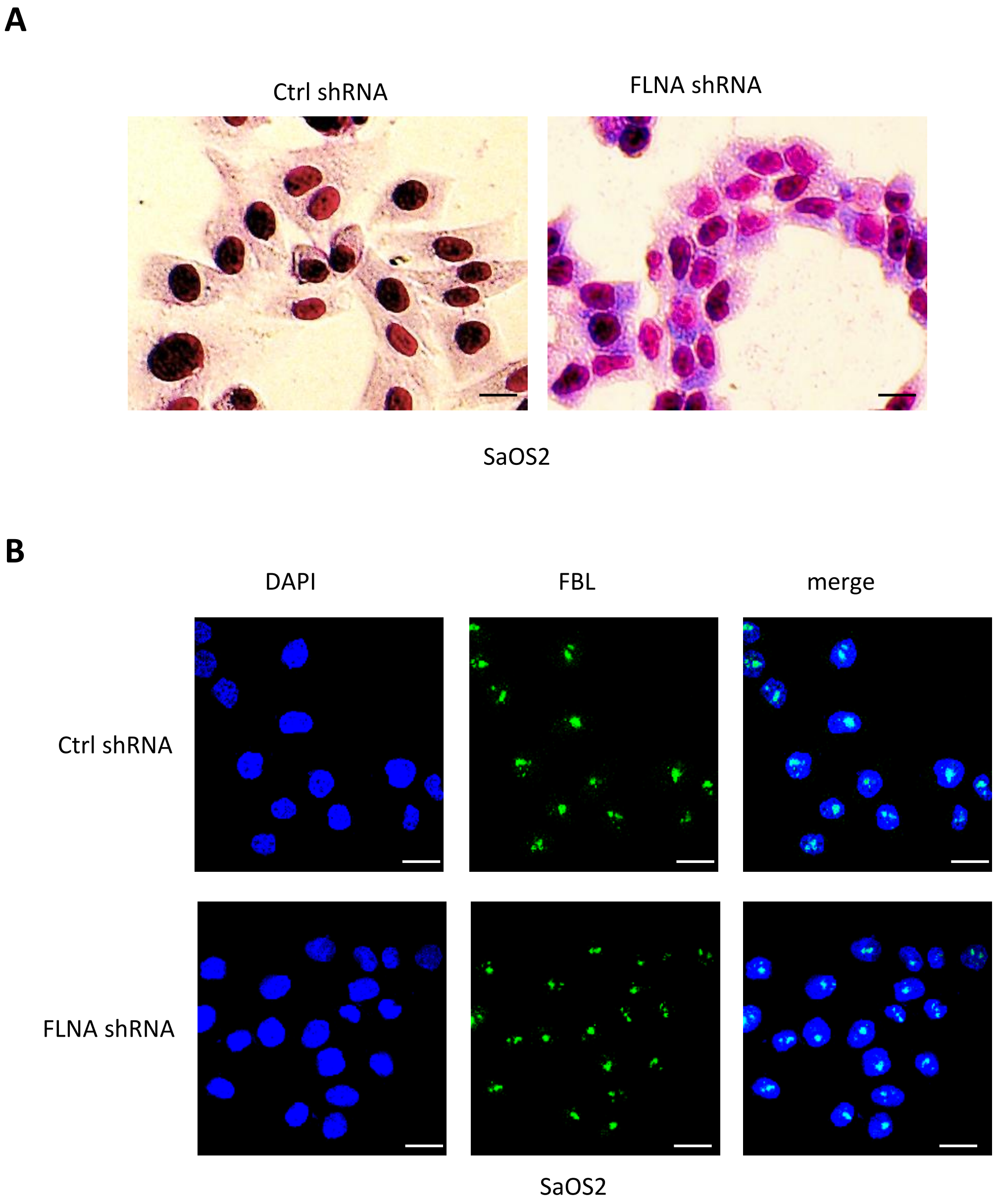

Fig. S1. Effect of Filamin A knockdown on the morphology of SaOS2 cells. (A) SaOS2 cells cultured in 12-well plates were stained by and imaged under a bright field microscope. Scale bars in the images represent 50 µm. (B) SaOS2 cells grown on coverslips were used for immunofluorescence assays with an anti-Fibrillarin (a nucleolar protein marker) antibody, followed by staining with Dapi. The resulting samples were imaged under a confocal fluorescence microscope. Scale bars in the images represent 5 µm.

Fig S2

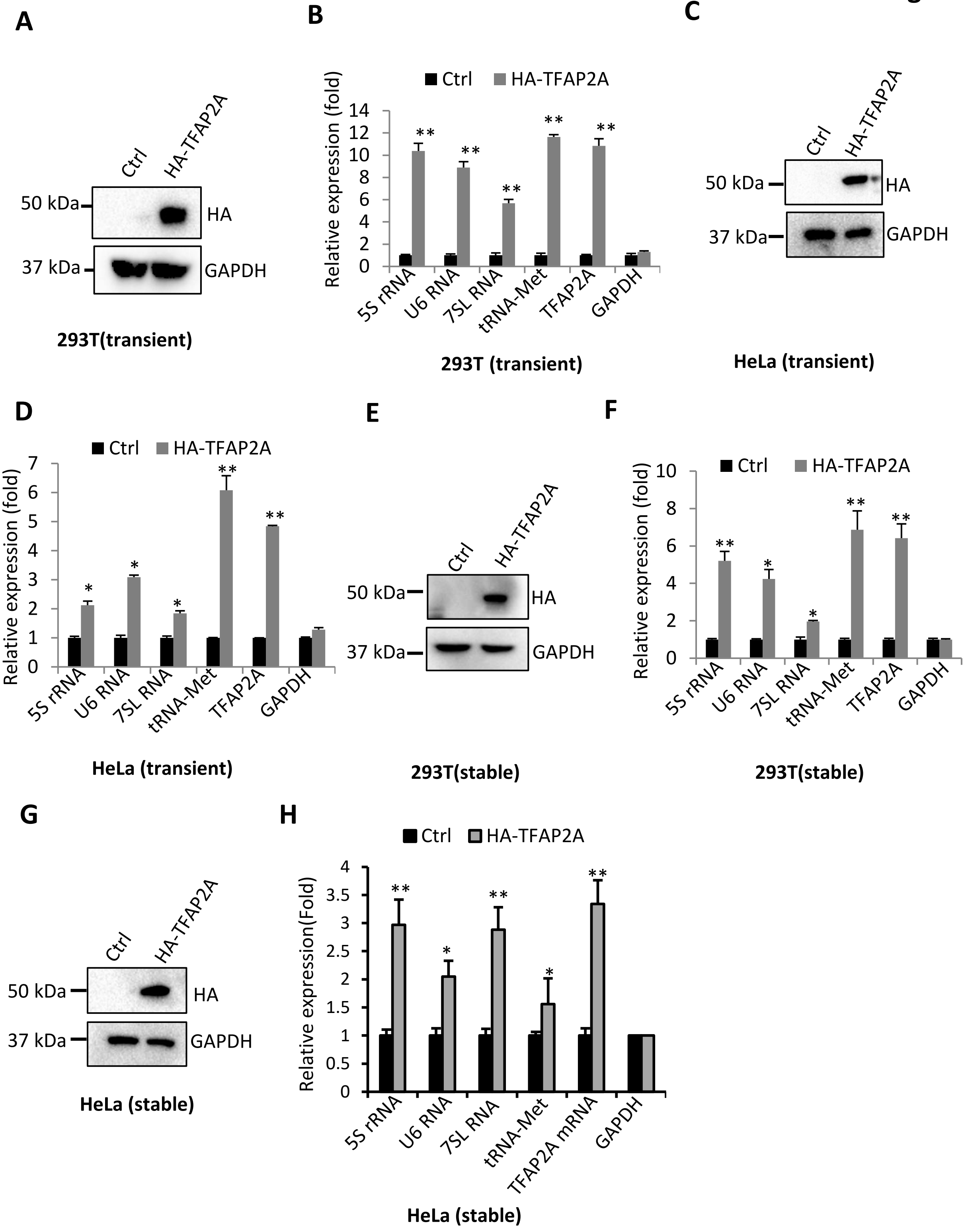

A
Ctrl
HA-TFAP2A
50 kDa
HA
37 kDa
GAPDH
293T(transient)
B
Ctrl
HA-TFAP2A
Relative expression (fold)
14
12
10
8
6
4
2
0
**
5S rRNA
U6 RNA
7SL RNA
tRNA-Met
TFAP2A
GAPDH
293T (transient)
C
Ctrl
HA-TFAP2A
50 kDa
HA
37 kDa
GAPDH
HeLa (transient)
D
Ctrl
HA-TFAP2A
Relative expression (fold)
7
6
5
4
3
2
1
0
*
**
5S rRNA
U6 RNA
7SL RNA
tRNA-Met
TFAP2A
GAPDH
HeLa (transient)
E
Ctrl
HA-TFAP2A
50 kDa
HA
37 kDa
GAPDH
293T(stable)
F
Ctrl
HA-TFAP2A
Relative expression (fold)
10
8
6
4
2
0
5S rRNA
U6 RNA
7SL RNA
tRNA-Met
TFAP2A
GAPDH
293T(stable)
G
Ctrl
HA-TFAP2A
50 kDa
HA
37 kDa
GAPDH
HeLa (stable)
H
Ctrl
HA-TFAP2A
Relative expression(Fold)
4
3.5
3
2.5
2
1.5
1
0.5
0
5S rRNA
U6 RNA
7SL RNA
tRNA-Met
TFAP2A mRNA
GAPDH
HeLa (stable)

Fig. S2. TFAP2A overexpression stimulated expression of Pol III products. (A, B) Transient expression of HA-TFAP2A in 293T cells increased the expression of Pol III products. HA-TFAP2A and Pol III products were respectively analysed by Western blot and RT-qPCR after transient transfection of the vectors expressing HA-TFAP2A or the empty vectors (control) into 293T cells. (C, D) Transient expression of HA-TFAP2A in HeLa cells enhanced the expression of Pol III products. HA-TFAP2A and Pol III products were respectively detected by Western blot and RT-qPCR after transient transfection of the vectors expressing HA-TFAP2A or the empty vectors (control) into HeLa cells. (E, F) Stable expression of HA-TFAP2A in 293T cells augmented Pol III product expression. HA-TFAP2A and Pol III products were respectively analysed by Western blot and RT-qPCR using a 293T cell line stably expressing HA-TFAP2A and its control cell line. (G, H) Stable expression of HA-TFAP2A in HeLa cells activated the expression of Pol III products. HA-TFAP2A and Pol III products were respectively analysed by Western blot and RT-qPCR using a HeLa cell line stably expressing HA-TFAP2A and its control cell line. Each column in histograms represents the mean ± SD of three biological replicates. *, $p<0.05$; **, $p<0.01$. *P* values were obtained by Student's *t* test.

Fig S3

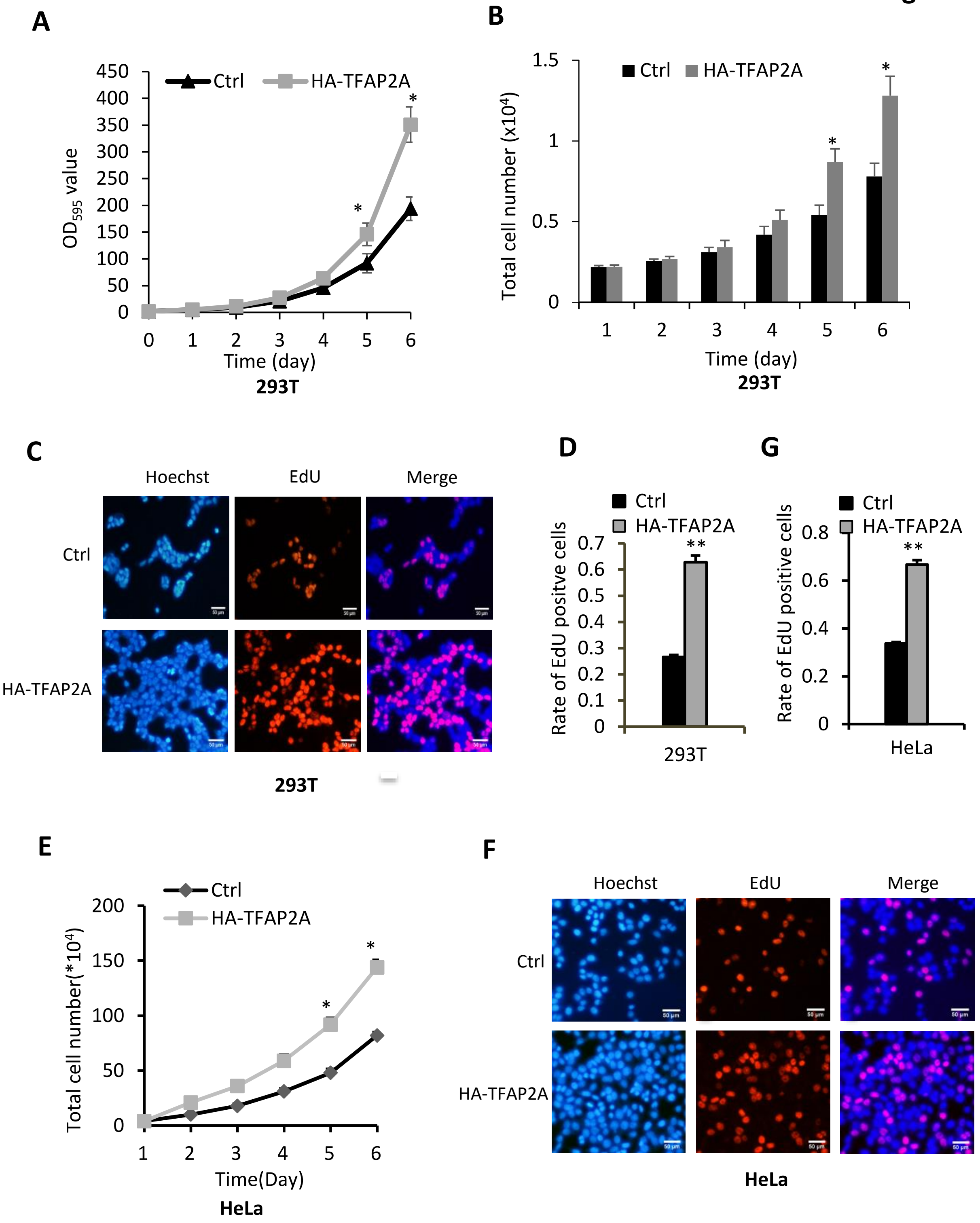
A
Ctrl
HA-TFAP2A
OD595 value
Time (day)
293T
B
Ctrl
HA-TFAP2A
Total cell number (x10⁴)
Time (day)
293T
C
Hoechst
EdU
Merge
Ctrl
HA-TFAP2A
293T
D
Ctrl
HA-TFAP2A
Rate of EdU positve cells
293T
G
Ctrl
HA-TFAP2A
Rate of EdU positive cells
HeLa
E
Ctrl
HA-TFAP2A
Total cell number(*10⁴)
Time(Day)
HeLa
F
Hoechst
EdU
Merge
Ctrl
HA-TFAP2A
HeLa

Fig. S3. **Overexpression of TFAP2A promoted cell proliferation .** (A, B) Overexpression of TFAP2A increased 293T cell proliferation activity. A 293T cells line expressing HA-TFAP2A or its control cell line were cultured in 12-well or 96-well plates, cell proliferative activity was monitored every 24 hours by cell counting (A) and MTT assays (B). (C, D) The results of EdU assays for 293T cell lines with TFAP2A overexpression. A 293T cells line expressing HA-TFAP2A or its control cell line were cultured in 12-well plates. After 24h, cells were labeled with EdU for 2 hours, then subjected to staining with an EdU detection kit and imaging under a fluorescent microscope . The scale bars in images (C) represent 50 μm. The rate of positive cells were analyzed statistically (D). (E-G) Overexpression of TFAP2A increased the proliferative activity of HeLa cells. Cell counting (E) and EdU assays (F,G) for HeLa cells were performed as described for 293T cells. The scale bars in images (F) represent 50 μm. Each column in histograms represents the mean ± SD of three biological replicates. *, $p<0.05$; **, $p<0.01$. *P* values were obtained by Student's *t* test (D and G) or two-way ANOVA (A, B and E).

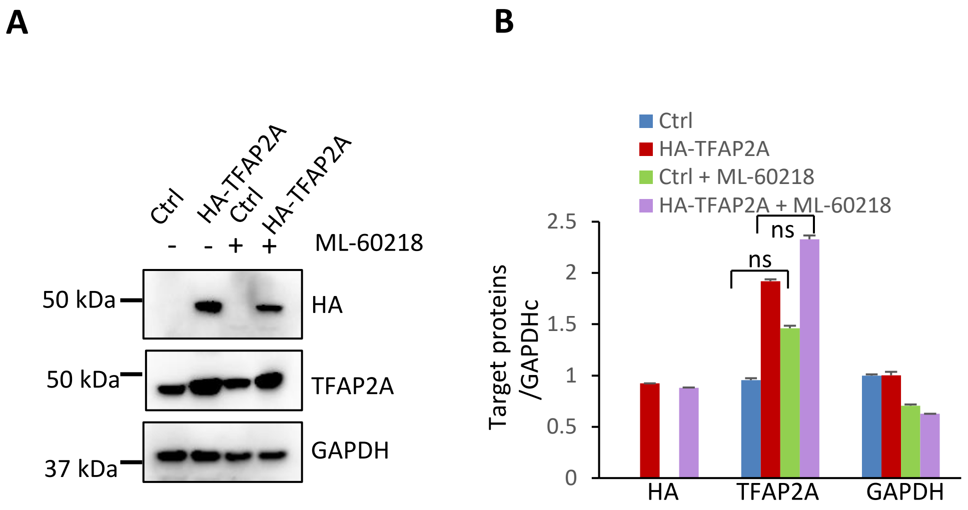

Fig **S4 Effect of ML-60218 treatment on the expression of TFAP2A in 293T cells.** Western blot (**A**) was performed using a 293T cell line expressing HA-TFAP2A and its control cell line treated with or without ML-60218 (54 µM), the resulting images were quantified by ImageJ software (**B**). ns: not significant.

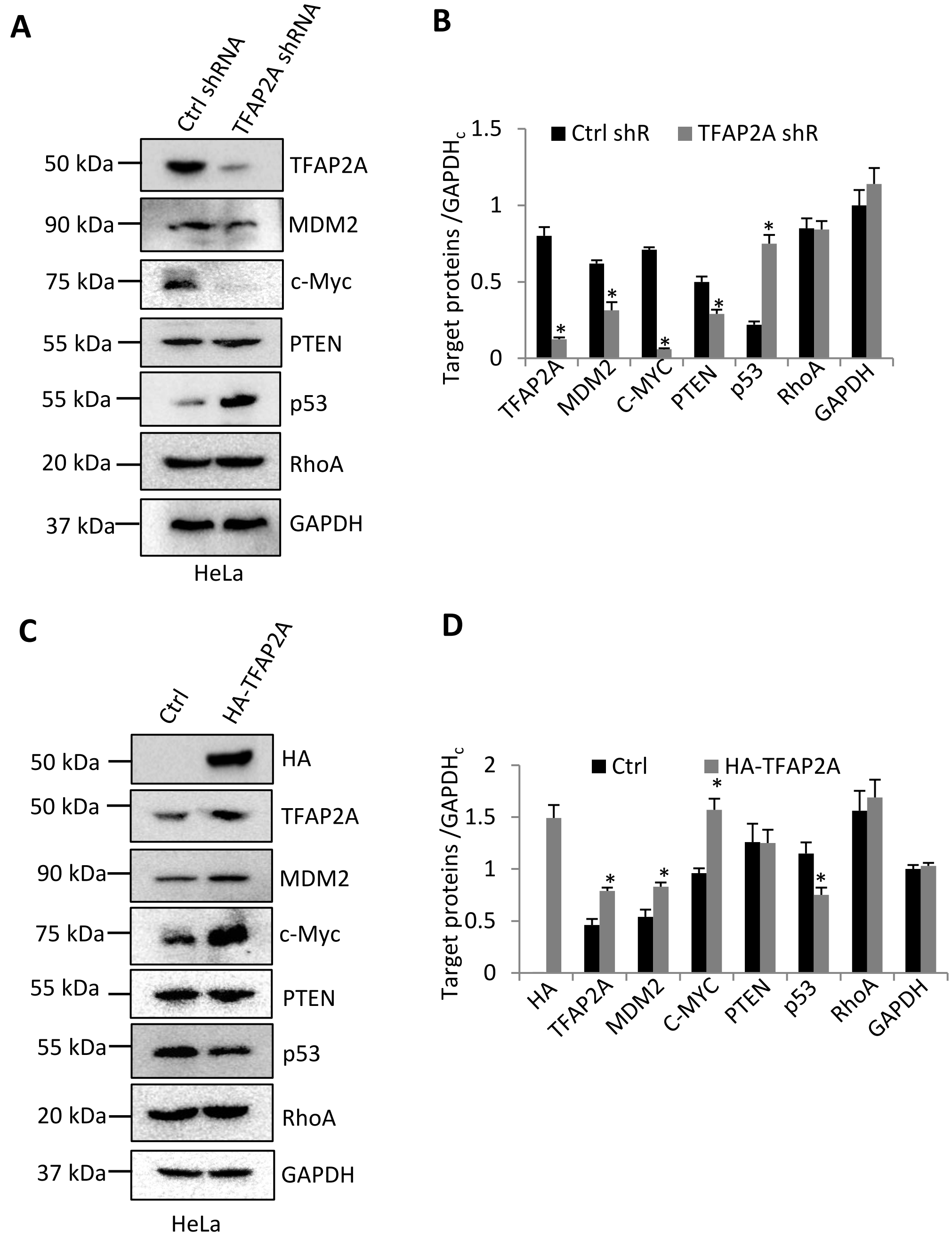

Fig S5. The effect of TFAP2A knockdown and overexpression on the expression of oncogenic factors, tumor suppressors and signaling factors. (**A**,**B**) Western blot showing the effect of TFAP2A silencing on the expression of oncogenic factors, tumor repressors and signaling factors in HeLa cells. B represents the quantified result of the blots obtained in A. GAPDHc: GAPDH for control group. (**C**, **D**) Western blot showing the effect of TFAP2A overexpression on the expression of oncogenic factors, tumor repressors and signaling factors in HeLa cells. D represents the quantified result of the blots obtained in C. GAPDHc: GAPDH for control group. *, Each column in histograms represents the mean±SD of three replicates. *p*<0.05; **, *p*<0.01. *P* values were obtained by Student's *t* test.

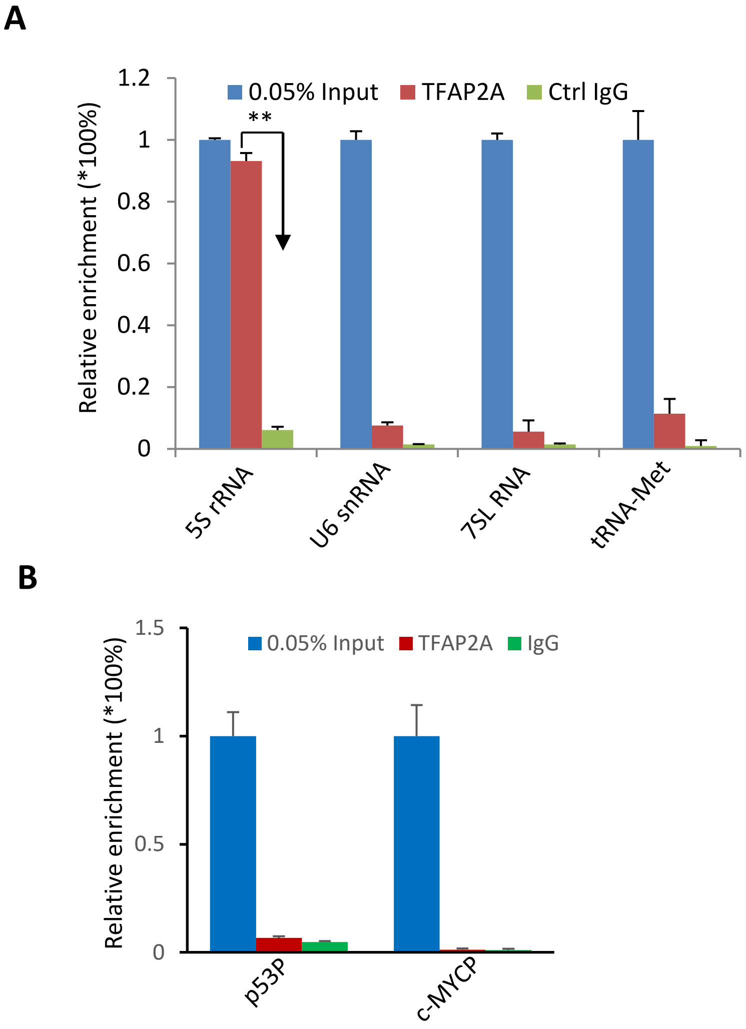

Fig S6. Analysis of TFAP2A occupancy at Pol III target loci and the promoters of *p53* and *c-MYC genes*. (A) ChIP qPCR results showing TFAP2A occupancy at Pol III target loci. (B) ChIP qPCR results showing TFAP2A occupancy at promoters of *p53* (*p53*P) and *c-MYC (c-MYC*P*)* genes. Each column in graphs represents the mean±SD of three biological replicates. **, $p<0.01$. *P* values were obtained by Student's *t* test

Fig. S7

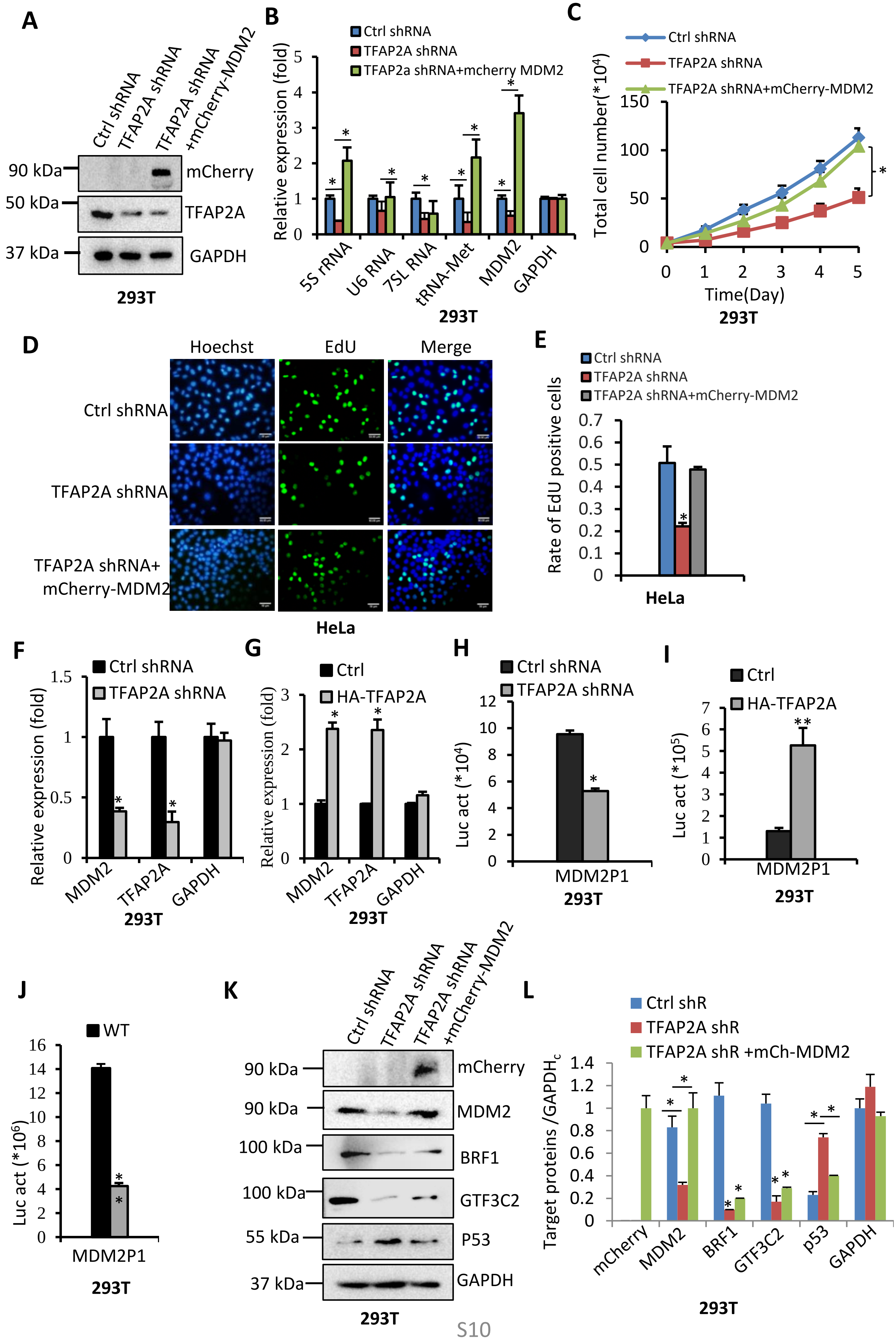
A
Ctrl shRNA
TFAP2A shRNA
TFAP2A shRNA +mCherry-MDM2
90 kDa
50 kDa
37 kDa
mCherry
TFAP2A
GAPDH
293T
B
Ctrl shRNA
TFAP2A shRNA
TFAP2a shRNA+mcherry MDM2
Relative expression (fold)
5S rRNA
U6 RNA
7SL RNA
tRNA-Met
MDM2
GAPDH
293T
C
Ctrl shRNA
TFAP2A shRNA
TFAP2A shRNA+mCherry-MDM2
Total cell number(*10⁴)
Time(Day)
293T
D
Hoechst
EdU
Merge
Ctrl shRNA
TFAP2A shRNA
TFAP2A shRNA+ mCherry-MDM2
HeLa
E
Ctrl shRNA
TFAP2A shRNA
TFAP2A shRNA+mCherry-MDM2
Rate of EdU positive cells
HeLa
F
Ctrl shRNA
TFAP2A shRNA
Relative expression (fold)
MDM2
TFAP2A
GAPDH
293T
G
Ctrl
HA-TFAP2A
Relative expression (fold)
MDM2
TFAP2A
GAPDH
293T
H
Ctrl shRNA
TFAP2A shRNA
Luc act (*10⁴)
MDM2P1
293T
I
Ctrl
HA-TFAP2A
Luc act (*10⁵)
MDM2P1
293T
J
WT
Luc act (*10⁶)
MDM2P1
293T
K
Ctrl shRNA
TFAP2A shRNA
TFAP2A shRNA +mCherry-MDM2
90 kDa
90 kDa
100 kDa
100 kDa
55 kDa
37 kDa
mCherry
MDM2
BRF1
GTF3C2
P53
GAPDH
293T
L
Ctrl shR
TFAP2A shR
TFAP2A shR +mCh-MDM2
Target proteins /GAPDH
mCherry
MDM2
BRF1
GTF3C2
p53
GAPDH
293T

Fig S7. TFAP2A modulates Pol III-directed transcription by affecting the activity of a MDM2/P53 pathway. (A) Western blot showing the expression of mCherry-MDM2 and TFAP2A in 293 cell lines stably expressing TFAP2A shRNA or control shRNA or both of TFAP2A shRNA and mCherry-MDM2. (B) mCherry-MDM2 expression reversed the inhibition of Pol III–directed transcription caused by TFAP2A silencing. RT-qPCR was performed using the RNA extracted from the cell line as indicated. (C)) mCherry-MDM2 expression rescued the inhibition of 293T cell proliferation caused by TFAP2A silencing. Cell counting was performed at different time points using cell line as indicated. (D ,E) EdU assays showing the effect of mCHerry-MDM2 expression on HeLa cell lines expressing TFAP2A shRNA. EdU was used to label HeLa cell lines for 2 hours. After staining, cell samples were imaged under a fluorescent microscope (D) and subjected to statistical analysis (E). The scale bars in images (D) represent 50 µm. (F, G) The effect of TFAP2A expression alteration on MDM2 RNA expression. RT-qPCR was performed using the RNA extracted from 293T cell lines with TFAP2A silencing (F) or overexpression (G). (H, I) Reporter assays showing the effect of TFAP2A silencing (H) or overexpression (I) on the activity of the MDM2 promoter 1. (J) Mutations of TFAP2A consensus sequences in the MDM2 promoter reduced the MDM2P1 activity. Luciferase assays were performed using 293T cell lines transiently transfected with the promoter-driving reporter vectors. (K,L) mCherry-MDM2 expression inhibited the activation of p53 expression caused by TFAP2A silencing. Western blot was performed using the cell lysate and the antibodies against the proteins as indicated. L represents the quantification of the blots obtained in K. $GAPDH_c$: GAPDH for control group. *, p<0.05; **, p<0.01. P values were obtained by Student's t test.

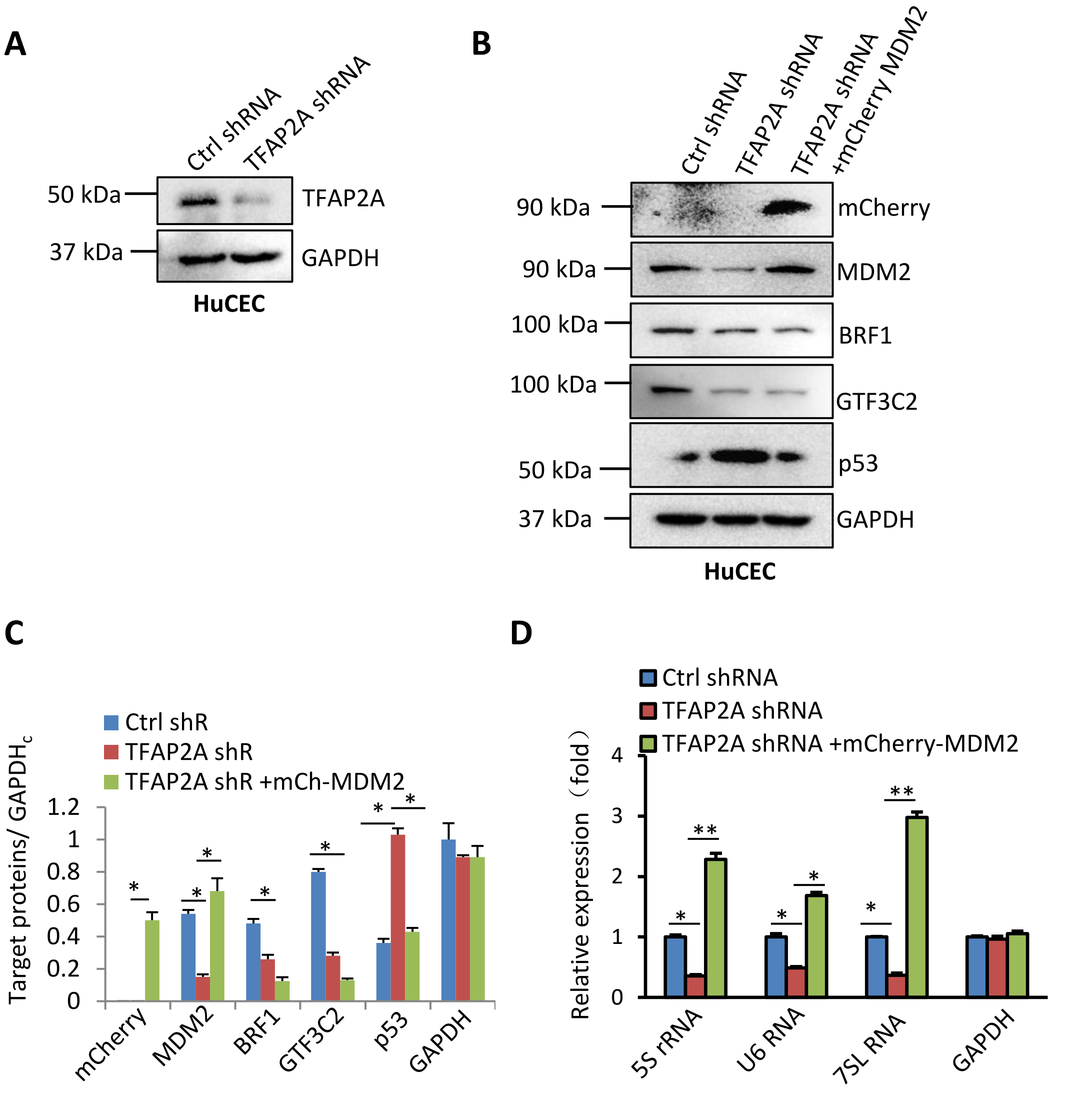

Fig S8. MDM2 overexpression reversed the inhibition of Pol III–directed transcription mediated by TFAP2A silencing in human cervical epithelial cells. (A) Western blot results showing the generation of human cervical epithelial cells (HuCEC) stably expressing TFAP2A shRNA or control shRNA. (B, C) Western blot results showing the generation of HuCEC expressing both TFAP2A shRNA and mCherry-MDM2 and the effect of mCherry-MDM2 expression on the expression of BRF1, GTF3C2 and p53. C represents the quantified result of the blots obtained in B. GAPDHc: GAPDH for control group. (D) mCherry-MDM2 expression reversed the inhibition of pol III-directed transcription mediated by TFAP2A silencing. Each column in histograms represents the mean±SD of three biological replicates. *, *p*<0.05; **, *p*<0.01. *P* values were obtained by Student's *t* test.

Fig S9

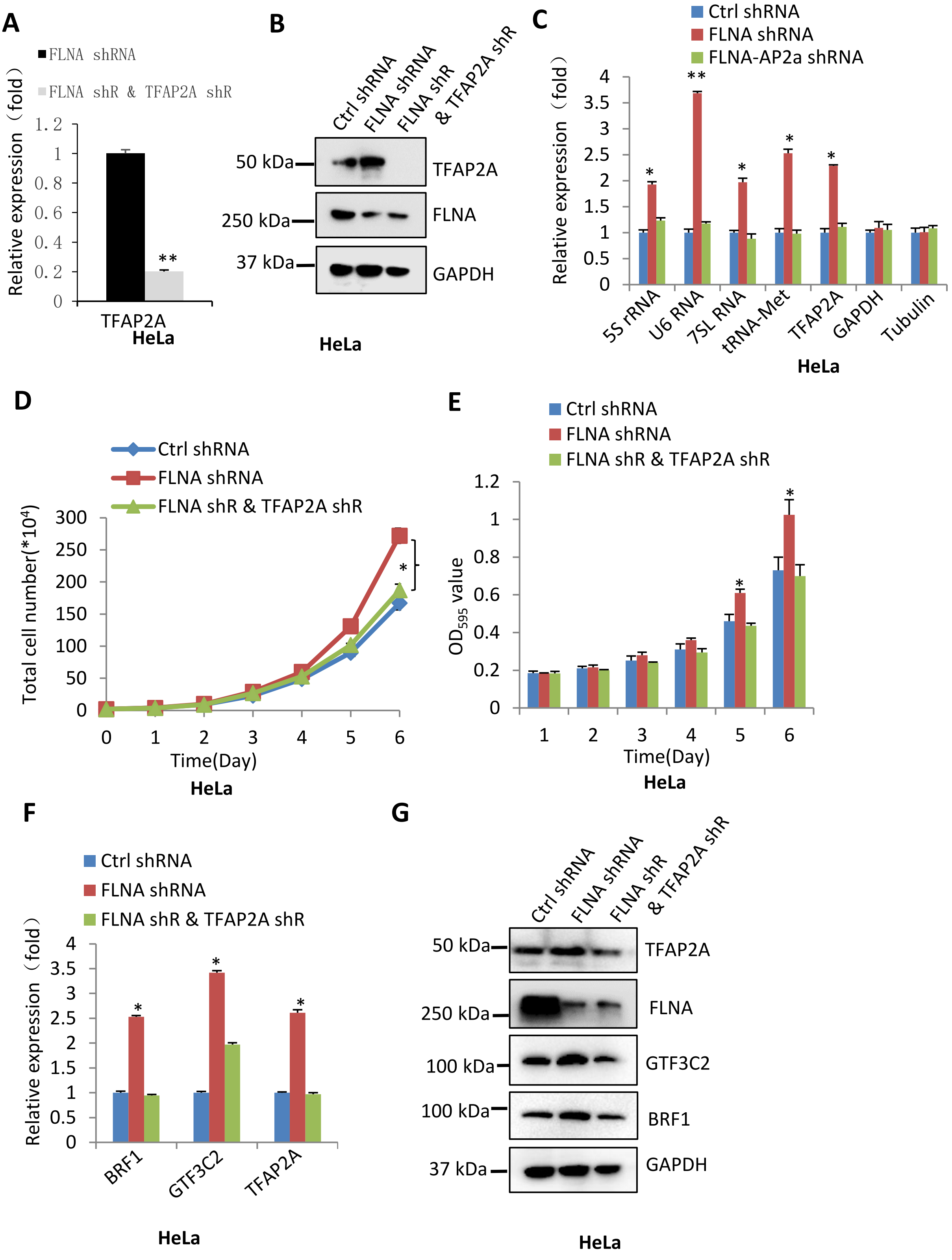
A
FLNA shRNA
FLNA shR & TFAP2A shR
Relative expression（fold）
1.2
1
0.8
0.6
0.4
0.2
0
**
TFAP2A
HeLa
B
Ctrl shRNA
FLNA shRNA
FLNA shR & TFAP2A shR
50 kDa
TFAP2A
250 kDa
FLNA
37 kDa
GAPDH
HeLa
C
Ctrl shRNA
FLNA shRNA
FLNA-AP2a shRNA
Relative expression（fold）
4
3.5
3
2.5
2
1.5
1
0.5
0
5S rRNA
U6 RNA
7SL RNA
tRNA-Met
TFAP2A
GAPDH
Tubulin
HeLa
D
Ctrl shRNA
FLNA shRNA
FLNA shR & TFAP2A shR
Total cell number(*10^4)
300
250
200
150
100
50
0
0 1 2 3 4 5 6
Time(Day)
HeLa
E
Ctrl shRNA
FLNA shRNA
FLNA shR & TFAP2A shR
OD595 value
1.2
1
0.8
0.6
0.4
0.2
0
1 2 3 4 5 6
Time(Day)
HeLa
F
Ctrl shRNA
FLNA shRNA
FLNA shR & TFAP2A shR
Relative expression（fold）
4
3.5
3
2.5
2
1.5
1
0.5
0
BRF1
GTF3C2
TFAP2A
HeLa
G
Ctrl shRNA
FLNA shRNA
FLNA shR & TFAP2A shR
50 kDa
TFAP2A
250 kDa
FLNA
100 kDa
GTF3C2
100 kDa
BRF1
37 kDa
GAPDH
HeLa

Fig S9. TFAP2A is required for the activation of Pol III-directed transcription induced by FLNA downregulation. (A, B) Generation of the HeLa cell line stably expressing both FLNA shRNA and TFAP2A shRNA. TFAP2A expression was detected by RT-qPCR (A) and Western blot (B) using HeLa lines as indicated. (C) TFAP2A silencing inhibited the activation of Pol III-directed transcription induced by FLNA silencing. (D, E) TFAP2A depletion reduced the increase of proliferative activity caused by FLNA silencing. Cell counting (D) and MTT assays (E) were performed using HeLa cell lines as indicated. (F, G) TFAP2A silencing inhibited the activation of BRF1 and GTF3C2 expression caused by FLNA downregulation. Expression of BRF1 and GTF3C2 was performed by RT-qPCR (F) and Western blot (G) using HeLa cell lines as indicated. Each column in histograms represents the mean ± SD of three biological replicates. *, $p<0.05$; **, $p<0.01$. *P* values were obtained by Student's *t* test (A, C and F) or two-way ANOVA (D and E).
